# Exploring the Virome of Nile Tilapia (*Oreochromis niloticus*) Using Metagenomic Analysis

**DOI:** 10.3390/pathogens14090935

**Published:** 2025-09-16

**Authors:** Amira Ezzat, Ahmed Abd El Wahed, Arianna Ceruti, Amel M. El Asely, Mohamed Shawky Khalifa, Andrew D. Winters, Uwe Truyen, Adel A. Shaheen, Mohamed Faisal

**Affiliations:** 1 Department of Aquatic Animal Medicine, Faculty of Veterinary Medicine, Benha University, Toukh 13736, Egypt; amira.alghanam@fvtm.bu.edu.eg (A.E.); amlvet@yahoo.com (A.M.E.A.); shaheen_aa@yahoo.com (A.A.S.); 2 Institute of Animal Hygiene and Veterinary Public Health, Faculty of Veterinary Medicine, University of Leipzig, An den Tierkliniken 43, D-04103 Leipzig, Germany; ahmed.abd_el_wahed@uni-leipzig.de (A.A.E.W.); arianna.ceruti@uni-leipzig.de (A.C.); truyen@vmf.uni-leipzig.de (U.T.); 3 Department of Fisheries and Wildlife, College of Agriculture and Natural Resources, Michigan State University, East Lansing, MI 48824, USA; khalifa9@msu.edu; 4 Department of Physiology, School of Medicine, Wayne State University, 656 West Kirby Street, Detroit, MI 48202, USA; awinters@med.wayne.edu; 5 Department of Pathobiology and Diagnostic Investigation, College of Veterinary Medicine, Michigan State University, East Lansing, MI 48824, USA

**Keywords:** Nile tilapia, virome, Oxford nanopore sequencing, family *Amnoonviridae*, order Articulavirus, metagenomics

## Abstract

Nile tilapia (*Oreochromis niloticus*) is an indispensable source of high-quality protein worldwide. Along with the exponential expansion of tilapia aquaculture, several novel pathogenic viruses have emerged, and some cause significant economic losses. Unfortunately, there is scarce information on the biology and epidemiology of these viruses. This exploratory metagenomic study used Oxford Nanopore Technology (ONT) sequencing to profile the virome compositions of both wild and farmed Nile tilapia across five regions in Egypt. The Nile tilapia virome was dominated by two double-stranded DNA bacteriophages, *Muvirus mu* and *M. sfmu*, which constituted 79.8% of the detected sequences. Eukaryotic viruses, including members of the families *Amnoonviridae*, *Peribunyaviridae*, and *Baculoviridae*, were also identified. Two giant DNA viruses known to infect *Acanthamoeba* spp., *Mollivirus* sp., and *Pandoravirus* sp. were identified in the spleen virome of tilapia from a single sampling site. The diversity analysis showed no significant differences among tissue types or sampling sites. Phylogenetic analyses were performed on a single virus detected of potential pathogenicity, an amnoonvirus. The analyses demonstrated that the detected virus is a member of the family *Amnoonviridae* and placed it alongside members of the *Tilapinevirus* genus. The virus, however, was distinct from the other two members in the genus: *T. tilapae* and *T. poikilos*. This study underscores the usefulness of ONT in providing a foundational understanding of the Nile tilapia virome.

## 1. Introduction

For as long as historical archives have been kept, people residing along the banks of the River Nile have been praising Nile tilapia (*Oreochromis niloticus*; families: Cichlidae; Osteichthyes) as their preferred edible fish. Currently, the aquaculture of this species is one of the fastest growing globally. As per the United Nations Food and Agriculture Organization, Nile tilapia farming helps support local economies and livelihoods, especially in rural areas of developing countries [1,2]. Apart from its global aquaculture importance, in natural ecosystems, Nile tilapia plays a vital role in nutrient cycling and aquatic vegetation control, matters that are essential to maintain the fragile balance of the aquatic environment [3].

Concomitant with the global expansion of Nile tilapia farming, novel viruses have emerged that are ravaging both wild and farmed tilapia species [4,5]. For example, tilapia lake virus (TiLV) was first detected in 2009 [6] and has since left a trail of mortality episodes in wild and farmed tilapia in the Americas, Asia, and Africa [7]. In 2022, the World Organization for Animal Health (WOAH) listed this disease as one of its reportable infections. This enveloped, single-stranded, negative-sense RNA virus has puzzled scientists regarding its unique biological characteristics, mutations, reassortment formation, and unsettled taxonomy [8]. While the segmented nature of TiLV RNA and the conserved sequences at the 5′ and 3′ ends of its RNA segments on both sides suggest its inclusion in the family *Orthomyxoviridae*, TiLV exhibits some unique features that no other orthomyxovirus has. TiLV RNA is divided into 10 segments, which exceeds the numbers of segments (six–eight segments) in other orthomyxoviruses [9]. Moreover, unlike other orthomyxoviruses, TiLV does not agglutinate piscine, avian, or mammalian red blood cells [10], and the majority of its encoded proteins have no homology with other viral or cellular proteins, except the ORF encoded by segment 1, which has weak sequence homology to the influenza C virus PB1 subunit [11,12]. To settle the taxonomy of this emerging virus, a novel family was established in 2017: the family *Amnoonviridae* [13] under the order Articulovirales [14], which also encompasses the family *Orthomyxoviridae*. As it currently stands, *Amnoonviridae* contains a single genus: the *Tilapinevirus*, with a single species, *T. tilapiae*, and a single member, the tilapia lake virus (TiLV) [13]. However, this taxonomy is being revisited due to the genetic variations among TiLV isolates and the increasing proposals to add more genera and species to the family *Amnoonviridae.*

Identifying the composition of the bacterial community associated with Nile tilapia and the factors that may lead to future potential outbreaks are the subjects of several studies [15,16,17]. The information generated from these studies has shaped the aquaculture practices of this species and guided the design of biosecurity protocols [17,18]. On the contrary, no thorough study has ever been conducted to determine the viral community (virome) associated with tilapia. Considering the uninterrupted emergence of novel tilapia-pathogenic viruses, belonging to a variety of virus taxonomic orders and families, identifying the tilapia virome components becomes a priority. In other marine and freshwater fish species, the virome composition has been studied [19,20,21], and viruses of potential pathogenicity to fish have been identified, even those that cannot replicate in vitro [19,22,23,24,25,26,27].

Metagenomic high-throughput sequencing platforms have emerged as the method of choice to investigate the compositions of viral communities associated with living organisms, as they overcome the absence of phylogenetic markers in viruses [28]. Among the commonly used methods to determine virome richness and diversity, Oxford Nanopore Technology (ONT) seems to be superior in terms of its ability to sequence relatively large reads in real time and to allow the detection of rare and low-abundance taxa [22], an advantage of ONT that is vital for the precise identification of core virome components. To this end, an exploratory metagenomic study using the ONT platform was initiated with two objectives. The first objective was to identify the main components of the Nile tilapia virome. The second objective was to shed light on the viruses present in its virome that are of potential pathogenicity to Nile tilapia. Information to be gained from this study can improve the understanding of the intricacies between aquatic viruses and Nile tilapia in its native environment.

## 2. Materials and Methods

### 2.1. Ethics Statement

The handling, euthanasia, and sample collection were performed according to the protocols approved by the Institutional Animal Care and Use Committee Research Ethics Board, Faculty of Veterinary Medicine, Benha University, Egypt (Ethical Number: BUFVTM56-11-23, approved on 16 July 2023).

### 2.2. Fish Sample Collection

**Figure 1:**
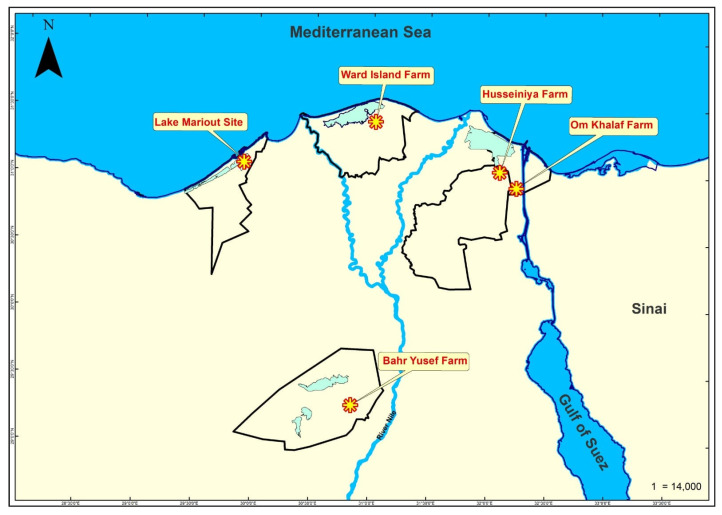
Map of Northern Egypt showing the five sampling sites where the Nile tilapia was collected in this study.

The initial study to determine the virome composition of Nile tilapia (*Oreochromis niloticus*) was performed using fish collected from a farm in Al-Husseiniya District (31°3′30.481″ N and 32°6′20.346″ E), Alsharqia Governorate, Egypt (referred to here as Husseiniya Farm). Five fish were collected randomly (142.2 ± 29.11 g in weight, 9.2 ± 0.91 cm in fork length) for use in the analysis. Additional samples of tilapia organs were collected from four other sites and included in the analyses. Sampling sites are displayed in Figure 1. Free-ranging fish (n = 5) were collected from Lake Mariout (31°6′28.227″ N and 29°53′11.639″ E). The fish measured 7.9 ± 0.74 cm in fork length and weighed 21.0 ± 1.46 g. Another sample of 3 fish (548.0 ± 79.18 g and 25.1 ± 1.52 cm) was collected from the Ward Island Farm, Kafr El-Sheikh Governorate (31°25′38.811″ N and 31°1′24.798″ E), which will be referred to as Ward Island. The third sample of 3 fish (218.0 ± 63.01 g, 21.9 ± 2.86 cm) was obtained from Om Khalaf Farm, Port Said Governorate (30°56′23.243″ N and 32°15′3.170″ E), and it will be referred to as Om Khalaf. The last sample of 2 Nile tilapia (162.5 ± 53.03 g, 18.5 ± 2.12 cm) was collected from Bahr Yusef Farm, Faiyum Governorate (29°18′27.855″ N and 30°50′45.447″ E), which will be referred to as Bahr Yusef. Samples from the 5 sites were collected between the months August and October 2023.

The collected fish were euthanized by hypothermia and immediately transported on ice to the laboratory at the Department of Aquatic Animal Medicine, Benha University, Moshtohor, Qalyubia Governorate, Egypt, where they were processed upon receipt. The fish exhibited no behavioral abnormalities; however, a few fish exhibited mild general disease signs, such as scale loss and caudal fin erosion. Wild fish collected from Lake Mariout were obviously emaciated compared to the farmed cohorts.

In the laboratory, samples from liver, kidney, spleen, and brain were aseptically collected from individual fish. In addition, gills were sampled from fish collected from Husseiniya and Bahr Yusef farms. All collected samples were immersed in RNAlater^®^ (Thermo Fisher Scientific, Waltham, MA, USA) and stored at −80 °C until further processing. The number of fish obtained from each sampling site, type of tissue collected, and number of pools prepared for metagenomic analysis following RNA or DNA extraction are displayed in Supplemental Table S1.

### 2.3. Nucleic Acid Extraction and Reverse Transcription

Nucleic acids were extracted from pooled samples collected from the organs of Nile tilapia: totals of 27 pools for RNA and 19 pools for DNA. The total RNA from 30 mg of pooled-sample homogenate was extracted using the RNeasy Mini Kit (Qiagen, Hilden, Germany) according to the manufacturer’s instructions. Eluted RNA samples were kept at −80 °C until use. The quantification of the extracted RNA was conducted with the Qubit™ RNA BR Assay Kit. RNA samples were reverse-transcribed into cDNA using the RevertAid First Strand cDNA Synthesis Kit (Thermo Fisher Scientific Inc.) according to the manufacturer’s instructions. After incubating with the mastermix for 10 min at 25 °C, reverse transcription was conducted for 60 min at 42 °C, followed by reverse transcriptase inactivation for 5 min at 70 °C. These cDNA samples were quantified using the Qubit™ dsDNA BR Assay Kit (Invitrogen, Thermo Fisher Scientific) and were stored at −20 °C till further processing.

DNA was extracted from pooled tissue samples using the DNeasy Blood and Tissue kit (Qiagen) according to the manufacturer’s instructions. All the extracted samples were quantified using a NanoDrop™ 2000/2000c Spectrophotometer (Thermo Fisher Scientific).

### 2.4. Oxford Nanopore Technology (ONT) Sequencing and Data Analysis

The barcoding and sequencing of both DNA and cDNA libraries were performed following the protocol of rapid sequencing gDNA barcoding for the Rapid Barcoding Kit 96 (SQK-RBK 110.96, Oxford Nanopore Technologies, Oxford, UK) and were purified using AMPure XP magnetic beads (Beckman Coulter GmbH, Krefeld, Germany). The library was sequenced for 72 h using a Spot-ON Flow Cell R9 version and the MinION Mk1C sequencing device (MinKNOW v21.11.6). The priming kit (EXP-FLP002) was prepared following the manufacturer’s protocol and was used to flush the flow cell. After sequencing for 72 h, basecalling of the fast5 raw files was performed using Guppy v6.5.7. Following sequencing, the workflow of Oxford Nanopore “What’s in My Pot Program (WIMP)” from the platform EPI2ME vesion v5.1.3 (Oxford Nanopore Technologies) was used to analyze the passed FASTQ files of successful reads. Geneious Prime^®^ 2023.2.1 was used to perform offline analysis, and the reads were screened using BLAST+ 2.17.0 against the NCBI virus database. Viruses were recognized based on their closest match in GenBank, with updated information on the virus taxonomy derived from the publications of the International Committee on the Taxonomy of Viruses (ICTV). Viruses known to not infect any fish species or members of viral genera or families not likely to infect fish (e.g., bacteriophages or insect viruses) were recorded as components of the Nile tilapia virome but were not included in the phylogenetic or genetic analyses performed on sequences of viruses of potential pathogenicity to fish. These viruses were co-assembled, and their contigs of nucleotide and amino acid sequences were subjected to additional phylogenetic analyses, as described below.

### 2.5. Virome Diversity and Statistical Analysis

The relative viral abundances across sampling sites and organs were calculated using reads per kilobase (RPK), where

RPK = (number of reads mapped to a viral sequence)/ (length of the viral sequence in kilobases).

The percentage of relative abundance was calculated by dividing each RPK value by the sum of all RPK values within the virome and multiplying by 100:

Relative abundance (%) = (RPK/ΣRPK) × 100.

The virome diversity across the different sampling locations and organs was assessed by calculating the alpha diversity, using both the observed virome richness (the number of detected viral species per organ or site) and the Shannon index. The Shannon index was calculated according to the formula *H* = −∑ (*pi lnpi*), using the cumulative read counts for each viral species obtained from ONT sequencing data, analyzed using the EPI2ME platform. Calculations were performed using Microsoft Excel, where *pi* was the proportional abundance of each virus species in the samples. The proportional abundance was calculated by dividing the number of cumulative reads of each virus species in all samples by the total number of cumulative reads of all viruses detected in each sample.

For the statistical analysis, samples from Om Khalaf were excluded, as they were processed for RNA extraction only. We tested for the significance differences among the four sampling regions (Husseiniya, Bahr Yusef, Ward Island, and Lake Mariout) and different organs (gills, brain, liver, spleen, and kidneys) in the viral abundance, richness, and diversity using the Kruskal–Wallis non-parametric test, where the significance levels were calculated using the Monte Carlo simulation of 10,000 samples. Moreover, a chi-square test for differences in the medians was also performed according to the above settings.

### 2.6. Further Characterization of Detected Amnoonvirus

More than 200 reads (228 reads from 31 Nile tilapia organ-pooled samples) identified nucleotide sequences of an amnoonvirus in Nile tilapia organs (referred to as AmnoonvirusEGY1). The numbers of reads varied from one RNA segment to another and were 69, 27, 18, 15, 44, 17, 8, 14, 8, and 8 for segments 1–10, respectively. The closest match to this virus in GenBank is the tilapia lake virus, a virus of high pathogenicity to tilapia species [29]. To confirm the identity of AmnoonvirusEGY1, a series of phylogenetic analyses on the obtained sequences were performed.

The software Porechop_ABI version 0.2.4 (https://github.com/bonsai-team/Porechop_ABI, accessed on 5 May 2024) was used to trim ligated sequencing adapters from the ends of Nanopore reads, as described by Bonenfant et al. [30]. All FASTQ files were processed using Porechop_ABI with default settings. Following trimming, Minimap2 version 11.1.0 was first used to map the reads to the tilapia lake virus reference genome assembly (GCF_001630085.1). Only the mapped reads were retained and merged into a single file for co-assembly using MEGAHIT version 11. MEGAHIT was chosen for its ultrafast performance in metagenomic assembly and its ability to leverage mapped reads from multiple samples to improve the assembly accuracy and contig length, making it particularly suitable for complex viral genomes [31].

Partial sequences of the AmnoonvirusEGY1 assembled contig of segments 1 (399 nt.), 5 (466 nt.), and 7 (316 nt.) were compared individually to representative members of the order Articulavirales, including unclassified proposed members of the family *Amnoonviridae*, the index strain of *Tilapinevirus* (TiLV), and several orthomyxoviruses (Table 1). The numbers of viruses included in the phylogenetic analyses varied from one segment to another depending on the availability of sequences in the public databases (accessed during the period between 5 May 2024 and 26 June 2025), with segment 1 sequences having the highest number of available sequences among members of the order Articulovirales. As a result, the segment 1 contig sequence of the AmnoonvirusEGY1 analysis included an assortment of sequences spanning a wide variety of virus species within the order. For the segment 5 phylogenetic analysis, coding sequences from 32 tilapia lake virus (TiLV) isolates, two TiLV-like strains from guppies (Maracas-2015-1 and Maracas-2015-2 [31]), and two fancy-tailed guppy virus (FTGV) strains (Guppy/95/10/82 and CobraB-2 [32]) were obtained from the NCBI GenBank database and aligned with the segment 5 contig sequence of AmnoonvirusEGY1 (466 nt.).

For the segment 7 phylogenetic analysis, coding sequences from 10 tilapia lake virus (TiLV) isolates and two TiLV-like strains from guppies (Maracas-2015-1 and Maracas-2015-2 [31]) were retrieved from the NCBI GenBank database. Only sequences with a query coverage of 65% or higher were included; as a result, only the 10 TiLV isolates and the 2 TiLV-like guppy strains were retained, while the fancy-tailed guppy virus (FTGV) strains were excluded for segment 7 but not for the other two segments. These sequences were aligned with the segment 7 contig sequence of AmnoonvirusEGY1 (316 nt.) of this study.

To evaluate similarities with isolates from neighboring countries, segments 1, 5, and 7 of AmnoonvirusEGY1 were compared to the corresponding segments of TiLV strains reported in Israel. Segments 1 and 7 were aligned with four strains, while segment 5 was compared with three strains. All reference sequences were sourced from the NCBI database.

Multiple sequence alignments were generated using the MUSCLE algorithm in MEGA version 12.0.11 [33]. Subsequent analyses in MEGA included determining the best-fit nucleotide substitution model and constructing maximum-likelihood (ML) phylogenetic trees with 1000 non-parametric bootstrap replicates. Pairwise nucleotide and amino acid distances were calculated using the p-distance model.

**Table 1:** Viruses included in the PB1 gene phylogenetic analysis, including information on their current ICTV classifications (families), virus names, geographic origins of detection or sample collections, target host species (and reservoir hosts, where applicable), and relevant references. They include members of the family *Amnoonviridae*, such as the newly identified AmnoonvirusEGY1 from Egypt (this study), as well as both classified and unclassified viruses within the order Articulavirales. Additionally, representative viruses from the family *Orthomyxoviridae* are listed to provide broader phylogenetic context. GenBank accession numbers are included for sequence reference.

Current ICTV Classification Family	Virus Name	Geographical Location of Occurrence or Sample Collection	Target Host (and Reservoir Host Where Applicable	References, GenBank Accession Number
*Amnoonviridae*	AmnoonvirusEGY1	Egypt	Tilapia	This Study
*Amnoonviridae/* *Tilapinevirus*	Tilapia Lake Virus (TiLV)	Israel	Tilapia	KU751814.1 [34]
*Amnoonviridae*	Flavolineata virus	Bass Strait, Tasmania, Australia	Yellow-striped leatherjacket, *Meuschenia* *flavolineata*	MW198700.1 [35]
*Amnoonviridae*	Dolomieu virus	Laurentian Great Lakes, USA	Smallmouth bass, *Micropterus* *dolomieu*	GDQU01066121.1 [35]
*Amnoonviridae*	Asotus virus 1	Japan	Amur catfish, *Silurus asotus*	GHGF01033499.1 [35]
*Amnoonviridae*	Asotus virus 2	Japan	Amur catfish, *Silurus asotus*	GHGF01016319.1 [35]
*Amnoonviridae*	Przewalskii virus	China	Lake Qinghai scaleless carp, *Gymnocypris* *przewalskii*	GHYJ01002273.1 [35]
*Amnoonviridae*	Stewartii virus	China	Cyprinid fish, *Oxygymnocypris stewartii*	GIBO01031171.1 [35]
*Amnoonviridae*	Namensis virus	China	Cyprinid fish, *Gymnocypris namensis*	GHYH01080462.1 [35]
*Amnoonviridae*	Tilapia lake virus-like virus	Caribbean	Guppy, *Poecilia reticulata*	BK063200.1 [31]
*Amnoonviridae*	Fancy-tailed guppy virus	USA	*Guppy*, *Poecilia reticulata*	PP409995.1 [32]
Unclassified Articulavirales	Lauta virus	Australia	Gulf tree gehyra, *Gehyra lauta*	MT386081.1 [36]
*Orthomyxoviridae*	Infectious salmon anemia virus (ISAV)	Canada	Atlantic salmon, *Salmo salar*	NC_006503.1 [37]
*Orthomyxoviridae*	Yancheng orthomyxo-like virus	China	Branded goby, *Chaeturichthys* *stigmatias*	MG600035.1 [38]
*Orthomyxoviridae*	Rainbow trout orthomyxovirus-1	USA	Rainbow trout, *Oncorhynchus mykiss*	KX882062.1 [39]
*Orthomyxoviridae*	Pilchard orthomyxovirus	Australia	Atlantic salmon, *Salmo salar*	NC_078611.1 [40]
*Orthomyxoviridae*	Wuhan carp Isavirus 1	China	Goldfish, *Carassius auratus*	MG600055.1 [38]
*Orthomyxoviridae*	Wuhan spiny eel influenza virus	China	Lesser spiny eel, *Macrognathus* *aculeatus*	MG600038.1 [38]
*Orthomyxoviridae*	Wenling hagfish influenza virus	China	Inshore hagfish, *Eptatretus burgeri*	MG600051.1 [38]
*Orthomyxoviridae*	Wenling orthomyxo-like virus 2	China	Red spikefish *Triacanthodes* *anomalus*	MG600036.1 [38]
*Orthomyxoviridae*	Wuhan asiatic toad influenza virus	China	Asiatic toad, *Bufo gargarizans*	MG600045.1 [38]
*Orthomyxoviridae*	Xibalbanus thogotovirus 1	Australia	Cave swimmer, *Xibalbanus* *tulumensis*	BK067651.1 [41]
*Orthomyxoviridae*	Influenza A virus	USA	Blue-winged teal, *Anas discors*	KJ413483.1 [42]
*Orthomyxoviridae*	Influenza B virus	Canada	Egg-grown virus	NC_002204.1 [43]
*Orthomyxoviridae*	Influenza C virus	Japan	Homo sapiens	LC123402.1 [44]
*Orthomyxoviridae*	Influenza D virus	France	Bovine	LN559121.1 [45]

### 2.7. Concatenated Phylogenic Analysis Using Partial Nucleotide Sequences of Segments 1, 5, and 7 of Tilapinevirus spp. and AmnoonvirusEGY1

Partial nucleotide sequences corresponding to genomic segments 1, 5, and 7 of members of the family *Amnoonviridae* were individually aligned using Clustal in MEGA 6.0 with default parameters, and the resulting alignments were manually trimmed and concatenated to generate a final dataset comprising 1050 trimmed-nucleotide positions.

For the concatenated analysis, Bayesian phylogenetic inference was used, as it accommodates potential heterogeneity in evolutionary rates across sites and genomic segments, thereby allowing for the inference of relationships within the concatenated dataset. The General Time Reversible model with gamma-distributed rate variation among sites (GTR + G) was selected based on model testing in MEGA 6.0, and inference was performed using MrBayes v3.2.7a. Markov Chain Monte Carlo (MCMC) analysis was run for 1,000,000 generations using four chains, with sampling every 100 generations and the first 25% discarded as burn-in. Convergence was assessed based on the average standard deviation of split frequencies and potential scale reduction factors (PSRFs). Posterior probabilities ≥ 0.70 were considered to indicate strong clade support.

## 3. Results

### 3.1. Overview of Nile Tilapia Virome

**Figure 2:**
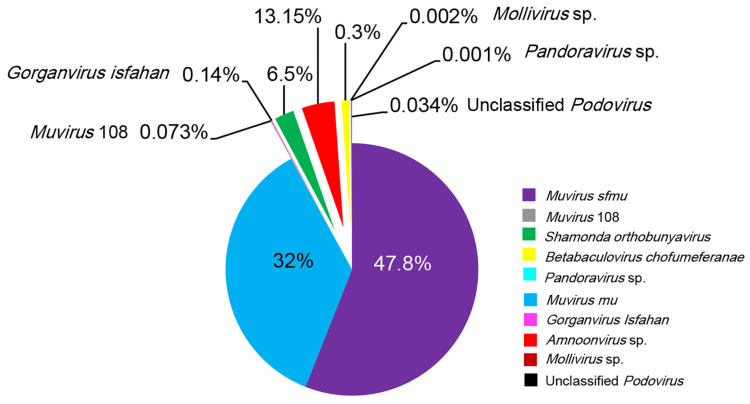
Pie chart showing percentage of relative abundance of each virus in the gills, brain, liver, kidneys, and spleen identified in 46 pooled samples of Nile tilapia collected from 5 sampling sites in Egypt.

Metagenomic ONT sequencing was performed on 46 tilapia sample pools. Assemblies from the metagenomes were screened and compared to NCBI GenBank and other databases. The majority of assemblies were recognized as bacteria (78%), archaea (<1%), and eukaryotes (21%), primarily fungi, while viruses accounted for only 1%. From the 46 pooled Nile tilapia tissue samples, taxonomic analyses determined the presence of eight DNA and two RNA viruses infecting bacteria, insects, amoeba, and fish (Figure 2). Due to the recent adoption by the ICTV of a new virus nomenclature protocol, the new nomenclature will replace previous ones with the old names mentioned in parentheses.

Two DNA bacteriophages dominated the tilapia: *Muvirus mu* (formerly known as *Escherichia virus Mu*) constituted ~32% of the virome, while *Muvirus sfmu* (formerly known as *Shiegella virus Sfmu*) constituted 47.8% of the virome in the 46 pooled samples. Both bacteriophages are members of the *genus Muvirus,* class *Caudoviricetes*. Reads of three other DNA bacteriophages were also identified in the tilapia virome, albeit at much lower relative abundances, namely, the transducing coliphage *Muvirus* 108 (~0.073%) (formerly known as *Escherichia* phage D108), *Gorganvirus isfahan* (0.14%) (formerly known as *Proteus virus Isfahan*), and an unclassified *Podovirus* sp. (0.034%) (now classified under the family *Autographviridae* [46]) (Figure 2).

The Nile tilapia virome also contained two viruses known to infect insects: Shamonda Orthobunyavirus and the DNA *Betabaculovirus chofumeferanae*. The Shamonda virus, which belongs to the Simbu group of the genus *Orthobunyavirus* of the family *Bunyaviridae*, constituted 6.5% of the tilapia virome. *B. Chofumeferanae*, originally known as *Choristoneura fumiferana granulovirus* (family: *Baculoviridae* [47]), constituted 0.3% of the tilapia virome. This virus is a double-stranded DNA that infects insects, mostly members of the orders lepidoptera and Hymenoptera (Figure 2). The two Acanthamoeba-infecting double-stranded DNA viruses belonging to the genera *Mollivirus* and *Pandoravirus* contributed 0.002% and 0.001% of the tilapia virome, respectively.

The only virus of interest for its potential pathogenicity to Nile tilapia detected was a single-stranded RNA virus with reads from 10 RNA segments, whose closest match was TiLV and which constituted 13.15% of the virome. Therefore, additional phylogenetic studies were performed to determine the precise taxonomy of this amnoonvirus, while no additional studies were performed on the other viruses detected in the Nile tilapia virome, as they are most likely of no hazard to fish.

### 3.2. Distribution of Viruses in Husseiniya Farm Nile Tilapia Organs

**Figure 3:**
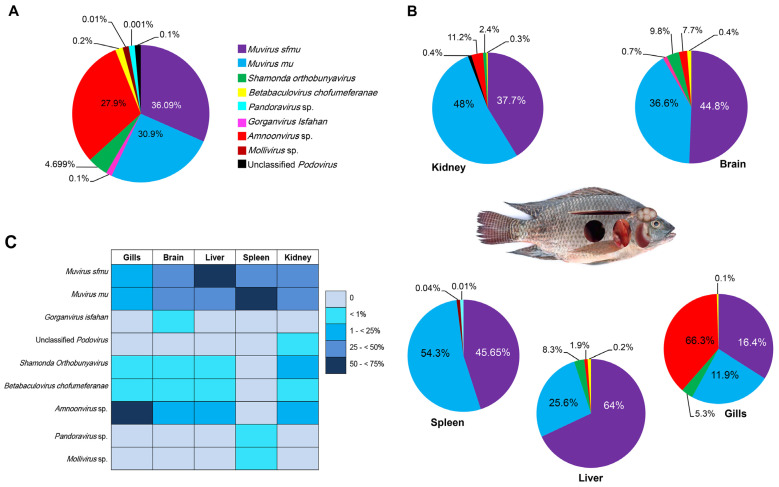
Relative abundances of viruses in organs of Husseiniya Farm Nile tilapia samples. (**A**) Pie chart showing abundance percentage of each virus in samples of five organs combined. (**B**) Pie charts showing variations in relative abundance percentages among organs. (**C**) Heat map showing uneven distributions and abundances of viruses in different organs.

The distribution analysis of viruses within the Nile tilapia organs was first performed at Husseiniya Farm, as it has a complete set of RNA- and DNA-extracted samples from each of the five organs examined in this study. As displayed in Figure 3A, nine viruses constituted the virome, of which four phages constituted 67.19% relative abundance in 19 sample pools from this farm, while the two insect viruses constituted 4.899%. The potentially fish-pathogenic amnoonvirus constituted a sizeable relative abundance percent in the tilapia virome with ~27.9% of the viruses. *Acanthamoeba* viruses contributed to 0.01% and 0.001% for *Mollivirus* sp. and *Pandoravirus* sp., respectively (Figure 3A).

The presence and relative abundances of the nine viruses varied greatly from one organ to another (Figure 3B,C). Two phages (*Muvirus sfmu* and *M. mu)* were present in the five organs examined. The highest relative abundance of *Muvirus sfmu* was in the liver, followed by the brain and spleen (*p* = 0.2), and the lowest prevalence of the virus was in the kidneys and gills. The *Muvirus mu* prevalence in the organs was a little different, with the highest diversity noticed in the spleen and kidneys, which was greater than that in the brain, although non-significant (*p* = 0.07), followed by the liver. The gills exhibited the lowest number of reads for the phages. The Shamonda virus prevalence was highly significant (*p <* 0.05) in both the brain and liver compared to the other organs (gills and kidneys). *Amnoonvirus* was detected in all organs except the spleen, with the highest relative abundance (*p* < 0.05) in the gills, followed by the kidneys, while the relative abundances of this virus were 7.7% in the brain sample and 1.9% in the liver sample. *Betabaculovirus chofumeferanae* was detected in all organs except the spleen, with nearly the same relative abundance. Four viruses were detected in samples of one organ only: *Gorganvirus isfahan* in the brain, *Podovirus* in the kidneys, and *Mollivirus* sp. and *Pandora* sp. in the spleen.

### 3.3. Comparison of Virome Compositions in Organs of Fish Collected from Five Sampling Sites

When data from all organs and sampling sites were put together, it became obvious that variations in the virome components exist among the organs and sampling sites. As displayed in Table 2, *Muvirus sfmu* and *Muvirus mu* were present in every organ of the fish collected from every sampling site. Both were the only viruses present in the Nile tilapia collected from Om Khalaf Farm. *Muvirus* 108 and *Gorganvirus isfahan* were confined to two sampling sites, while the two insect viruses were present in all sampling sites except Om Khalaf. On the contrary, the unclassified *Podovirus* and two *Acanthamoeba*-infecting viruses were detected at Husseiniya Farm only and not in fish from the other sample sites. The presence of the amnoonvirus was restricted to Husseiniya and Bahr Yusef only, though no major mortality episodes were reported from these two farms.

When the virome compositions were compared across different organs, variations were observed (Table 3); however, the overwhelming presence of the two dominant bacteriophages, *M. mu* and *M. sfmu*, rendered statistical analyses impractical for comparisons. Despite this, there were some interesting observations. For example, the gill virome of the fish from Husseiniya Farm was quite unique in its composition compared to all the other organs from the five sampling sites, with the amnoonvirus reads constituting ~66.3% of the total virome composition, while all other viruses were less represented (16.4, 11.9, 5.3, and 0.1% for *Muvirus sfmu*, *Muvirus mu*, Shamonda orthobunyavirus, and *Betabaculovirus chofumeferanae*, respectively). On the contrary, the samples of Bahr Yusef, whose gills were extracted for DNA only, contained the two dominant bacteriophages, *M. mu* and *M. sfmu*, only.

The brain virome was also heterogenous among the different sample sites, with no clear trend except the presence of *M. mu* and *M. sfmu* with an abundance of >70% in each sample (Table 3). The amnoonvirus was also present in the Husseiniya brain samples (7.7%) and was relatively high in the Bahr Yusef fish brain samples (31.3%). Other viruses were represented, albeit at low relative abundances, except the Shamonda Orthobunyavirus abundances, which were 24 and 33.6% in both the Bahr Yusef and Ward Island fish brains, respectively. A comparison between the Husseiniya Farm and Bahr Yusef brain samples proved non-significant (*p* = 0.18) (Table 3).

The liver virome was populated by the two dominant bacteriophages, *M. mu* and *M. sfmu*, with relative abundances of >89%. Only Husseiniya Farm tilapia livers harbored the amnoonvirus (1.9%) and the two insect viruses (8.5%). The same trend was noticed in the spleen viromes of the five sampling sites; i.e., *M. mu* and *M. sfmu* constituted > 90%.

The Husseiniya Farm spleen samples were the only samples in this study that contained the two *Acanthamoeba* viruses (<0.005%), while the Ward Island spleen samples harbored the two insect viruses (22%). The three kidney virome samples contained relatively high diversity despite the dominance of *M. mu* and *M. sfmu* occupying > 83% of the viromes. Amnoonvirus sequences were present in the kidneys of fish from both Husseiniya and Bahr Yusef farms (~11.2 and 10%, respectively), in addition to insect viruses and others (Table 3).

### 3.4. Virome Richness in Nile Tilapia Virome

The highest viral abundance was recorded at Husseiniya Farm, followed by Lake Mariout, Bahr Yusef, and Ward Island. Alpha diversity was applied to assess the virome diversity of the Nile tilapia across the different sampling sites and different organs, using both the observed virome richness (counting the number of detected viral species per organ and sampling site) and the Shannon index. As mentioned above, the number of Nile tilapia virome components are 10. Husseiniya Farm was the highest in viral richness (nine viruses), followed by Bahr Yusef (seven viruses), while the lowest richness was at Om Khalaf (two viruses) (Table 4). Statistical analysis revealed a significant difference among the regions in terms of the viral abundances (Kruskal–Wallis chi-squared = 7.14, df = 3, *p*-value = 0.03, which is significant). But there were no significant differences among the different regions in terms of viral richness (Kruskal–Wallis chi-square = 4.59, df = 3, *p*-value = 0.18).

Regarding the organs, the highest viral abundances were recorded in the brain samples, followed by the kidney and spleen samples, and then the gill and liver samples. The highest richness was observed in the brain and kidneys with seven viruses, followed by the spleen with six viruses, while the lowest numbers of viruses were recorded in the liver and gill samples, with a total of five virus species for each of them. The richness in any one organ sample never exceeded 6 (Table 4). Statistical analysis revealed a non-significant difference in the viral abundances among the organs (Kruskal–Wallis chi-squared = 2.6, df = 4, *p* = 0.71). In this respect, the virus richness (Kruskal–Wallis chi-square = 4.23, df = 4, *p* = 0.41) was also statistically non-significant.

### 3.5. Shannon Diversity Index of Nile Tilapia Virome

**Figure 4:**
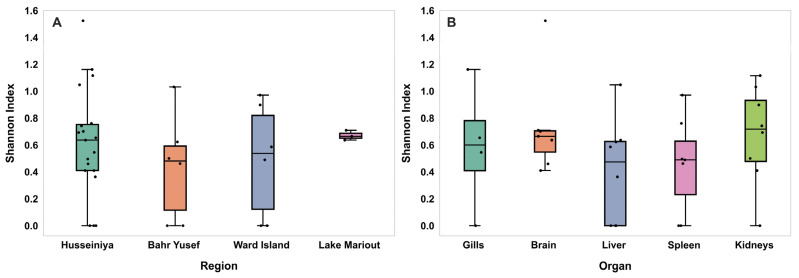
Dot-and-box plots showing virome diversity among different sampling regions and organs measured via the Shannon index. (**A**) The median values for all regions range from 0.48 to 0.66, with the lowest in Bahr Yusef and the highest in Lake Mariout. There were no significant differences. (**B**) The median values for all organs range from ~0.48 to 0.71, with the lowest in the liver and the highest in the kidneys. There were no significant differences.

The viral diversities measured using the Shannon index across the different sampling regions are presented in Figure 4A. The highest virome diversity was detected in Lake Mariout (H = 0.67), followed by Husseiniya (H = 0.62), Ward Island (H = 0.49), and then Bahr Yusef (H = 0.44). Despite this pattern, the observed differences were not statistically significant among the four regions (Kruskal–Wallis chi-squared = 1.82, df = 3, *p* = 0.62).

Regarding the virome diversity among the different organs, the Shannon index revealed distinct patterns of virome diversity among the examined fish organs (Figure 4B). The highest virome diversity was observed in the brain (H = 0.730), followed by the kidneys (H = 0.674), gills (H = 0.590), liver (H = 0.407), and spleen (H = 0.454). There were no statistically significant differences in the virome diversity measured via the Shannon index among the different organs (Kruskal–Wallis chi-square = 3.80, df = 4, *p* = 0.44).

### 3.6. Phylogenic Analysis of the Amnoonvirus

From the 31 pooled tilapia samples, 288 ONT sequencing reads were obtained whose closest match was TiLV. Phylogenetic and genetic analyses, which included several members of the order Articulovirales and the three contigs of AmnoonvirusEGY1 segments 1, 5, and 7, exhibited variations in both the nucleotide and amino acid sequences. However, the degree of divergence varied depending on the segment used and the number of closely related viruses whose sequences of segments 1, 5, and 7 were available in the public databases and were thus involved in the phylogenetic analysis.

**Figure 5:**
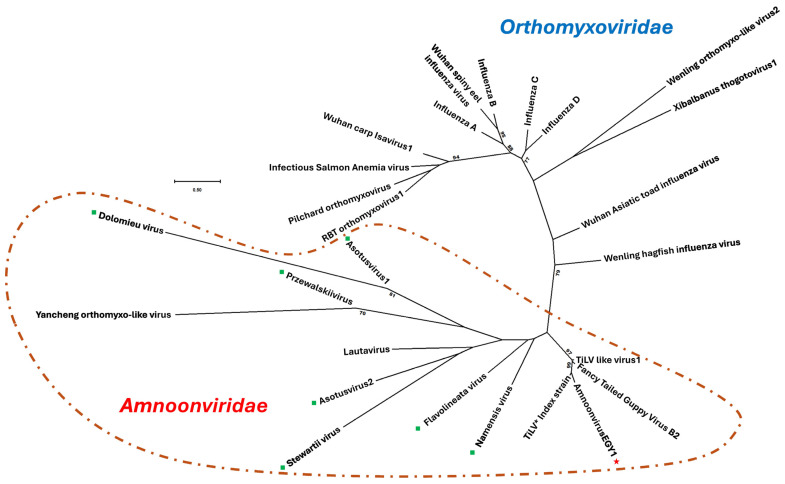
Unrooted maximum-likelihood phylogenetic tree based on PB1 gene nucleotide sequences showing the relationship of the newly identified AmnoonvirusEGY1 (red ★) to members of the order Articulavirales. AmnoonvirusEGY1 clusters with seven recently described, unclassified amnoonviruses (green squares: Asotus virus-1 and -2, Stewartii virus, Dolomieu virus, Przewalskii virus, Namensis virus, and Flavolineata virus) that share sequence similarity with segment 1. The tree highlights the close phylogenetic relationship between AmnoonvirusEGY1, unclassified amnoonviruses, and members of the family *Amnoonviridae*. RBT: Rainbow trout; the superscript black asterix ★ indicates TiLV index strain. Branch lengths represent the number of nucleotide substitutions per site, and bootstrap values ≥ 70% are indicated at the nodes.

For example, phylogenetic analysis based on the coding sequence of the PB1 gene (segment 1) revealed that AmnoonvirusEGY1 formed a well-supported basal branch within the order Articulavirales, clustering with members of the *Amnoonviridae* family (Figure 5).

Specifically, AmnoonvirusEGY1 grouped with members of the genus *Tilapinevirus*, showing a high bootstrap value of 99%. When compared directly with the TiLV index strain [6], eight unclassified amnoonviruses, TiLV-like strains, and the fancy-tailed guppy virus isolate [32], AmnoonvirusEGY1, formed a distinct well-supported basal branch with the TiLV index strain with a bootstrap value of 96%, further indicating its relatedness to the genus *Tilapinevirus tilapae*.

Further genetic analyses indicated that the partial PB1 gene (segment 1) of AmnoonvirusEGY1 displayed notable divergence from other members of the order Articulavirales. The pairwise nucleotide distances ranged from 13.38% (vs. TiLV index strain) to 78.32% (vs. *Dolomieu* virus, an unclassified *Amnoonviridae* member) (Supplemental Table S2). The amino acid distances were generally higher, ranging from 16.3% (vs. TiLV index strain) to 97.9% (vs. Wuhan spiny eel influenza virus, infectious salmon anemia virus, and pilchard orthomyxovirus) (Supplemental Table S3). These findings, in addition to the 10-segmented-RNA nature, support the classification of the Egyptian isolate within the *Amnoonviridae* family. Analysis within the *Amnoonviridae* family, specifically, demonstrated PB1 gene nucleotide distances ranging from 13.13% (vs. TiLV index strain) to 71.30% (vs. Asotus virus 2, an unclassified amnoonvirus) (Supplemental Table S4). The corresponding amino acid distances ranged from 19.19% (vs. TiLV index strain) to 92.86% (vs. Dolomieu virus, an unclassified *Amnoonviridae* member) (Supplemental Table S5). These results support that the AmnoonvirusEGY1 isolate is a member of the family *Amnoonviridae*, clustering most closely with the *Tilapinevirus* genus, despite the considerable genetic divergence, and it is distinctly far from the other unclassified members of the family *Amnoonviridae*.

**Figure 6:**
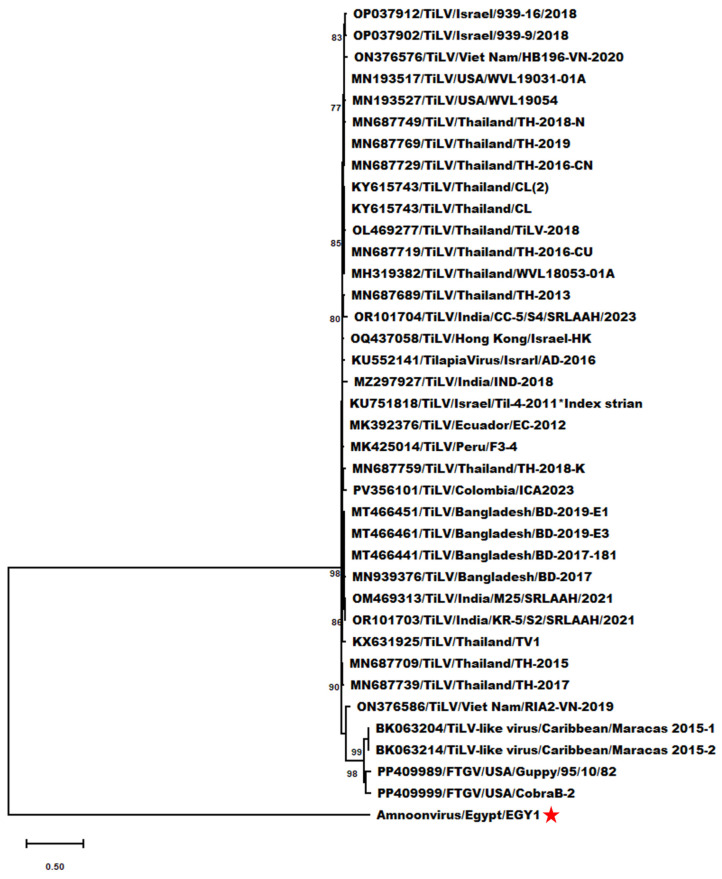
Maximum-likelihood phylogram depicting the relationships of Egyptian amnoonvirus (red ★) to 32 TiLV isolates, two TiLV-like strains from guppies, and two FTGV strains based on the nucleotide sequence alignment of segment 5. The included virus isolates are indicated by their NCBI GenBank accession numbers, virus names, countries, and isolates. Bootstrap values ≥ 70% are shown at the corresponding nodes. The scale represents the number of substitutions per site. The small superscript black ★ indicates the TiLV index strain according to ICTV.

In the same context, genetic analyses revealed that the partial segment 5 sequence of AmnoonvirusEGY1 exhibited notable genetic divergence from that of other members of the *Amnoonviridae* family (Figure 6). Nucleotide sequence differences ranged from 59.17% (compared to OM469313/TiLV/India/M25/SRLAAH/2021) to 61.98% (compared to the FTGV/Guppy95/10/82 strain), with 59.95% divergence from the TiLV index strain (Supplemental Table S6).

The amino acid sequence divergence was generally higher, ranging from 76.07% (vs. MZ297927/TiLV/India/IND-2018) to 77.50% (vs. two TiLV strains from Thailand—TV1 and TH-2018K, Accession Nos. KX631925 and MN687759—and two TiLV-like strains from guppies, Accession Nos. BK063204 and BK063214), with 76.67% divergence from the TiLV index strain (Supplemental Table S7).

**Figure 7:**
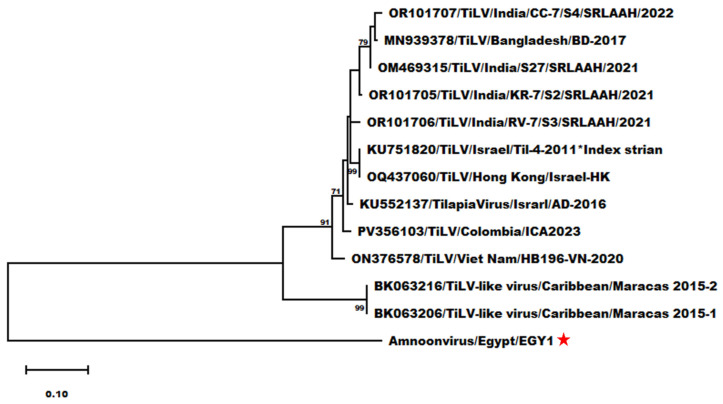
Maximum-likelihood phylogram depicting the relationships of Egyptian amnoonvirus (red ★) to 10 TiLV isolates and two TiLV-like strains from guppies based on the nucleotide sequence alignment of segment 7. The included virus isolates are indicated by their NCBI GenBank accession numbers, virus names, countries, and isolates. Bootstrap values ≥ 70% are shown at the corresponding nodes. The scale represents the number of substitutions per site. The small superscript black ★ indicates the TiLV index strain according to ICTV.

Additional genetic analyses using the partial segment 7 sequence of AmnoonvirusEGY1 also showed notable divergence from other members of the *Amnoonviridae* family (Figure 7). Nucleotide sequence differences ranged from 52.11% (compared to ON376578/TiLV/Viet_Nam/HB196-VN-2020) to 55.17% (compared to OM469315/TiLV/India/S27/SRLAAH/2021), with 53.26% divergence from the TiLV index strain (Supplemental Table S8).

The amino acid sequence divergence was generally higher, ranging from 62.03% (compared to multiple TiLV strains, including OR101705/TiLV/India/KR-7/S2/SRLAAH/2021; OQ437060/TiLV/Hong_Kong/Israel-HK; KU552137/TilapiaVirus/Israel/AD-2016; and ON376578/TiLV/Viet_Nam/HB196-VN-2020) to 65.82% (compared to two TiLV-like strains from guppies, Accession Nos. BK063204 and BK063214) (Supplemental Table S9). Regarding the question as to whether AmnoonvirsEGY1 has spread to other neighboring countries where Nile tilapia aquaculture is practiced, unrooted short trees constructed individually with each partial sequence of segments 1, 5, and 7 of the Egyptian strain versus the isolates from Israel listed in the NCBI database demonstrated that the Israeli strains tested are totally distinct from AmnoonvirusEGY1 (Supplemental Figure S1).

**Figure 8:**
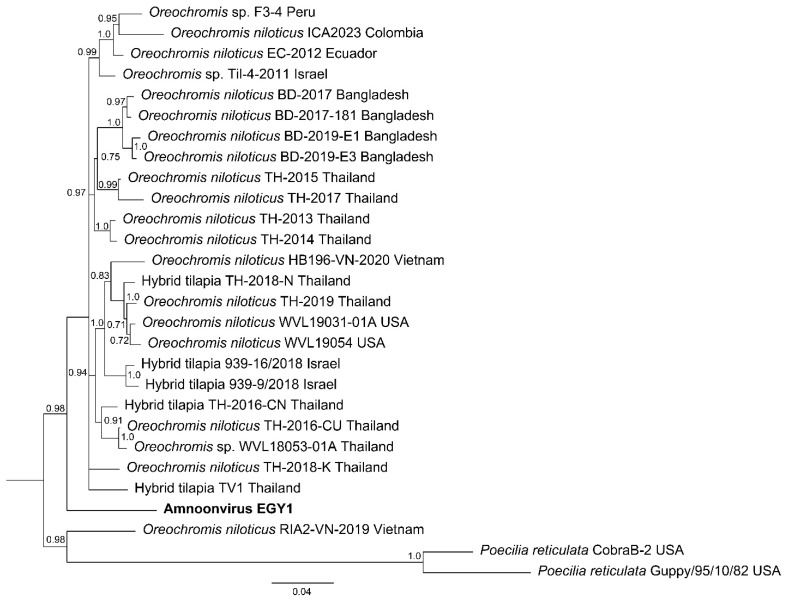
Bayesian phylogenetic tree inferred from concatenated partial genomic segments 1, 5, and 7 of *Amnoonviridae*. Phylogenetic inference was performed using the General Time Reversible (GTR) model with gamma-distributed rate variation among sites. Bayesian posterior probabilities > 0.70 are shown at the nodes. The final alignment included 1050 nucleotide positions. The scale bar represents the expected number of nucleotide substitutions per site.

Phylogenetic analysis of the concatenated partial sequences of segments 1, 5, and 7 placed AmnoonvirusEGY1 as a distinct lineage basal to the main clade of tilapia-associated amnoonviruses from Asia and the Americas (Figure 8). The Bayesian tree, inferred from a nucleotide alignment of concatenated partial genomic segments 1, 5, and 7, resolved multiple lineages within the family *Amnoonviridae*. AmnoonvirusEGY1 formed a distinct lineage, positioned as a sister to a strongly supported monophyletic group (posterior probability = 0.98) containing nearly all other known strains from *Oreochromis* and hybrid tilapia hosts sampled in Thailand, Vietnam, Bangladesh, Israel, Ecuador, Peru, and Colombia.

## 4. Discussion

### 4.1. Nile Tilapia Virome Overview

Findings of this study demonstrate that the Nile tilapia virome with 10 viruses is moderate in its richness when compared to that of viromes reported for other fish species. For example, 28 viruses constitute the viromes of free-ranging cyprinids [26], 52 viruses constitute those of wild-caught skipjack and yellowfin tuna [20], 20 viruses constitute those of wild-caught Atlantic horse mackerel, and 15 viruses constitute those of the gilthead seabream [48]. The presence of higher numbers of viruses in the viromes of wild freshwater and marine fish species may be due to the exposure of their wide geographical range to multiple aquatic organisms and ecosystems, as opposed to the Nile tilapia in this study, which were enclosed in earthen ponds or in the relatively small Lake Mariout. It is, however, noteworthy that, despite the inconsistency in the sample numbers and extraction methods (other than Husseiniya Farm tilapia), only minor variations in richness were noted.

Despite the descriptive nature of this study, it has generated multiple observations of interest and is worthy of follow-up studies. First, bacteriophages constituted the major component of the Nile tilapia virome. Sequence readings of bacteriophages were recorded in every sample analyzed, regardless of the sampling site or tissue of origin. This was not surprising, since bacteriophages are found in abundance in the aquatic environment [49,50]. It is strongly believed that bacteriophages in the aquatic environment are of paramount importance to controlling the species abundances of archaea and bacteria populations and thereby regulating several natural bioprocesses [49]. The presence of bacteriophages in fish viromes is also common [48,51], although their exact role in influencing any of the fish pathophysiological processes is currently unknown. The overwhelming relative abundances of bacteriophages in the Nile tilapia virome ranged from 56 to 100%, a matter that may have contributed to the non-statically significant differences in the comparisons performed in this study regarding the virome richness and composition in the different sampling sites and organs.

Since the ICTV adopted a new method of virus nomenclature and taxonomy [52], names of several viruses and families have changed, although not all public databases have been fully updated. For example, regarding the two dominant bacteriophages in this study, *Muvirus mu* was formerly known as *Escherichia virus Mu*, and *Muvirus sfmu* was formerly known as *Shigella virus Sfmu*; both are species in the genus *Muvirus*, class *Caudoviricetes*. Originally, the two bacteriophage species were placed in the family *Myoviridae,* order *Caudovirales*. This classification was based on their morphology; yet, full genome sequences of several myoviruses and subsequent phylogenetic analysis led to the abolition of the order *Caudovirales* with its three families, *Myoviridae*, *Podoviridae*, and *Siphoviridae*, and placed its genera and species directly under a new class: *Caudoviricetes* [53]. *Muvirus mu* and *Muvirus sfmu* are highly pathogenic to their primary hosts, *Escherichia coli* and *Shigella flexneri*, respectively [54]. Both *E. coli* and *Shigella flexneri* are known to infect fish, particularly those residing in chemically or biologically polluted waters, but rarely cause mortality episodes [55]. Therefore, the presence of bacteriophages specific to these two bacteria species was not unexpected, albeit not with such high frequencies, even in organs like the brain, whose blood barrier is difficult to overcome. However, using mammalian models, it was demonstrated that *Muvirus sfmu* can reach the brain within an hour of intraperitoneal administration [56]. This unique property of some bacteriophages makes them ideal candidates to deliver drugs to the central nervous system to treat diseases like Alzheimer’s disease and others [57].

The dominance of bacteriophages in the tilapia virome cannot be easily explained; however, several factors may have had an effect. One possibility is that following the demise of their hosts, the bacteriophages circulated in the blood in search of other susceptible bacteria, thereby minimizing the pathogen loads in the tilapia organs. Alternatively, infected bacteria may have fragmented the invading bacteriophage DNA through their well-developed Clustered Regularly Interspaced Short Palindromic Repeats (CRISPRs), causing viral DNA to reach other tissues [58,59].

Second, the Nile tilapia virome contained fewer reads of three additional host-specific bacteriophage species belonging to the abolished *Myoviridae* family. These are the transducing coliphage *Muvirus* 108 that causes chromosomal mutations in its host [60]; an unclassified *Podovirus* sp. (now classified under the family *Autographviridae*, [46]); and *Gorganvirus isfahan* (formerly known as *Proteus virus Isfahan*) [61]. The relatively low abundances of these three viruses and their unsettled taxonomies require additional investigation to determine whether they are a core component of the tilapia virome or were brought by some ephemeral bacteria [62]. Regardless of the mechanism by which bacteriophages dominate the viromes of Nile tilapia among other vertebrates, the influence of the bacteriophage presence requires additional studies [63].

Third, the detection of the giant viruses was surprising, since they have never been reported from Egypt before. The two giant Acanthamoeba-infecting DNA viruses, *Pandoravirus* sp. and *Mollivirus* sp., had the lowest relative abundances and distributions, being detected in spleen samples only and at one site. Most likely they were carried to tilapia by *Acanthamoeba* spp., which are ubiquitous in soil, freshwater, and marine habitats and often infect freshwater fish, including tilapia [64]. Several amoeba species have been described as infecting Nile tilapia in Lower Egypt, and they could be spreading these viruses [65]. Traditionally, giant DNA viruses were detected in remote marine sites or in the Arctic permafrost [66]; however, recent studies have demonstrated their presence in subarctic and temperate regions [67], and it is strongly believed that giant viruses are spreading worldwide through the ubiquitous nature of *Acanthamoeba* spp. in the environment. Regardless on how the two giant viruses find their way into tilapia tissues in Egypt, their potential effects on the aquatic environment and its fauna remain to be elucidated.

Fourth, the two insect viruses found in the Nile tilapia virome, *Betabaculovirus chofumeferanae* and *Shamonda orthobunyavirus*, may have found their way to tilapia through its feeding on zooplanktons, insects, and their larvae, and small crustacea, or through parasitism of tilapia by *Crustacean* and *Isopoda* spp. [68]. Neither of these insect viruses have been reported to cause diseases in Nile tilapia.

Last, only a single virus (out of 10) of potential pathogenicity to Nile tilapia was detected. There have been other viruses reported before from Nile tilapia in Egypt [e.g., viral nervous necrosis [69]; TiLV [70,71]], Africa [e.g., lymphocystis disease virus [72]; infectious pancreatic necrosis virus [73]; infectious spleen and kidney necrosis virus [74]], or from other nearby tilapia aquaculture facilities in Israel (e.g., the tilapia larvae encephalitis virus [75]). Other viruses have been reported to plague farmed tilapia in Asia, Europe, and Oceania [5].

The ONT sequence analysis identified reads in the tilapia virome whose closest matches were some sequences in each of the 10 RNA segments of tilapia lake virus (TiLV), a virus with a unique structure and biology that has baffled scientists since its emergence in 2009 [6]. The ONT sequence analysis indicated that we are dealing with a negative-sense, single-stranded, segmented RNA, which qualifies this virus to be considered as a member of the order Articulovirales. The order encompasses two families, *Orthomyxoviridae* and *Amnoonviridae*, and this virus genome seems to be formed of 10 RNA segments (based on the TiLV analogy), placing the virus in the family *Amnoonviridae* within that order. As per the ICTV [76], the family *Amnoonviridae* contains a single genus, *Tilapinevirus*, which contains a single species, *T. tilapiae*, which encompasses one member, TiLV. While this is the currently recognized classification of TiLV in the NCBI and other databases, there is an increasing number of proposals to modify this taxonomy at the species (TiLV), genus, and family levels.

At the TiLV level, studies demonstrate the continuous mutations and reassortant formation in the virus genome that led to the formation of genetic variants are circulating in the same country, on the same farm, or even in the same fish. These genetic variations among the increasing numbers of TiLV isolates have led scientists to wonder about the absence of the phylogenetic clustering of isolates originating from the same geographic location, as noticed in other RNA viruses, or why one TiLV in one country is genetically distantly related to all other TiLV strains from the same or other countries [7,8,77,78]. The pace of the genetic variability within TiLV isolates led Verma et al. [79] to conclude that TiLV genomic segments are being subjected to purifying selection, and that the emergence of genotypes and reassortant strains have become the dominant force in the evolution of TiLV and other members of the family *Amnoonviridae*.

At the *Tilapinevirus* genus level, there are proposals to include new species. Soto et al. [32] isolated an amnoonvirus from the fancy-tailed guppy (*Poecilia reticulata*), and, based on extensive phylogenetic and genetic analyses, the authors proposed a novel species in the genus *Tilapinevirus*, to be named *T. poikilos.* At the *Amnoonviridae* family level, there are a couple of proposals: one by Ortiz-Baez et al. [36], who proposed the Lautavirus (LATV) detected in the Australian gecko (*Gehyra lauta*) to form a novel genus within *Amnoonviridae*, and an additional study by Petrone et al. [41], which presented convincing arguments to the ICTV to relocate the genus *Isavirus,* currently in the family *Orthomyxoviridae*, to a genus in the family *Amnoonviridae*. Parallel to these two proposals, metatranscriptomic and data mining studies have revealed the presence of 12 hitherto divergent viruses that possess the characteristics of *Amnoonviridae* members. Additionally, homology to the mostly conserved heterotrimeric RNA-dependent RNA polymerase has been found, yet these viruses are distinct from members of the genus *Tilapinevirus* or any of the proposed species [21,35,38]. Considering the conundrum surrounding the taxonomy of the current and proposed members of the family *Amnoonviridae,* it was necessary to perform additional studies to shed light on the ten-segmented-RNA amnoonvirus detected in the Nile tilapia virome in this study, i.e., amnoonvirusEGY1.

### 4.2. Phylogenetic Studies on Sequences of AmnoonvirusEGY1

The three AmnoonvirusEGY1 contigs from segments 1, 5, and 7 were selected not only because of the relatively long length we were able to assemble but also because of the significance of segments 1 and 5 for the biology and taxonomy of amnoonviruses. In the family *Amnoonviridae*, segment 1 encodes the polymerase basic protein 1 (PB1) subunit of the heterotrimeric RNA-dependent RNA polymerase (RdRp). PB1 has been used to identify genetic diversity among TiLV isolates originating from several countries. Due to the absence of proofreading mechanisms during heterotrimeric RdRp gene replication, it is believed that this enzyme plays a major role in the evolution of RNA viruses, including TiLV [80], since the increased rates of mutations generate genetic variants, some of which can be selected under pressures imposed by the host immune response or strenuous ecological influences [81]. Despite this, Taengphu et al. [82] demonstrated robust homology in the PB1 nucleotides (95.0–99.94%) and amino acid sequences (99.00–100%) among TiLV isolates collected from several countries. On the contrary, our segment 1 phylogenetic analyses demonstrated that for AmnoonvirusEGY1, though it clustered with the family *Amnoonviridae* members, there was no homology as strong as that obtained among the TiLV isolates reported by Taengphu et al. [82]. Nucleotide sequence differences were the least with the TiLV index strain (~13%) and the guppy amnoonviruses (~22%) but were obviously distinct (>60% differences) from other orthomyxoviruses and unclassified amnoonviruses, with a similar trend noticed with amino acid sequences. These results demonstrate that AmnoonvirusEGY1 is a divergent member of the genus *Tilapinevirus* and is distant from the unclassified amnoonviruses.

A partial AmnoonEGY1 segment 5 sequence was used in the phylogenetic analyses with other *Tilapinevirus* spp. Segment 5 was selected due to its potential involvement in virus envelope formation. To determine the antigenicity of segment 5 polypeptides, Lueangyangyuen et al. [83] purified several polypeptide stretches and found that one of them, S5_196–272_, can mount a specific antibody response in tilapia if used as an immunogen. The phylogenetic analysis performed in this study with segment 5 could not include any of the 12 divergent, unclassified amnoonviruses because of the lack of this segment sequence in the public databases. The analysis demonstrated that the AmnoonvirusEGY1 partial sequence of segment 5 is very distinct from all the other viruses included in the analysis, and indeed, more so than what was noticed in the segment 1 phylogenetic analysis. This distinction increased with the amino acid sequences, suggesting that the mutations/substitutions were not silent, a matter that may affect the antigenicity of the strain if segment 5 polypeptides are used in vaccine preparation. In the same context, a substantial difference was noticed between TiLV isolates from tilapia and other *Tilapinevirus* spp. from guppies (>20%), as well as <6% variability among the various TiLV isolates used in this tree. These differences were not only in nucleotide sequences but also in amino acid sequences. The effects of these differences on the virus virulence and cross-reactivity among isolates need further investigation.

Like most TiLV segments, segment 7 encodes a protein of no homology to proteins of other viruses; however, this segment, along with segments 6 and 8, are phylogenetically close with gene sequences of different serotypes of the influenza virus [11,34]. The segment 7 phylogenetic tree obtained in this study echoed findings obtained with segment 5: TiLV originating from tilapia formed several clades with no distinct clustering for isolates obtained from the same geographical area, the *Tilapinevirus* spp. originating from guppies formed a separate clade, while the partial S7 sequence of AmnoonvirusEGY1 formed a separate branch.

It is currently unknown how widespread this divergent AmnoonvirusEGY1 is in the region. However, phylogenetic analyses performed on each AmnoonvirusEGY1 partial sequence of segments 1, 5, and 7 individually showed that the four Israeli isolates, whose sequences could be obtained from the public databases, were not identical at either the nucleotide or amino acid sequence levels to AmnoonEGY1. Unfortunately, no sequences of the TiLV isolates from Egypt, Lake Victoria [70,71,84], or other regions in Africa were available in the public databases for the segments used in this study. Therefore, more epidemiological studies are needed to determine the distribution of AmnoonvirusEGY1 in Africa. Also, the disjointed nature of the available sequences of the 12 unclassified divergent amnoonviruses makes precise positioning in phylogenetic analyses challenging.

The Bayesian tree, inferred from a nucleotide alignment of concatenated partial genomic segments 1, 5, and 7, resolved multiple lineages within the family *Amnoonviridae*. One of the hypotheses regarding the observed genetic heterogeneity among members of the *Tilapinevirus* genus is the reassortment events, particularly in segments 3 and 5 [77,78,79]. On the contrary, AmnoonvirusEGY1 formed a distinct lineage, positioned as a sister to a strongly supported monophyletic group (posterior probability = 0.98) containing nearly all *Tilapinevirus tilapae* isolated from *Oreochromis* spp. and other tilapia hosts and their hybrids sampled in Thailand, Vietnam, Bangladesh, Israel, Ecuador, Peru, and Colombia. The internal structure within this clade showed limited geographic clustering, suggesting historical dispersal via the trade of live fish and their products or shared ancestry. *Poecilia* (guppy) strains formed a separate, highly divergent lineage with long branch lengths, consistent with host-associated divergence. The phylogenetic placement of AmnoonvirusEGY1 suggests a distinct evolutionary history relative to previously characterized strains within the family *Amnoonviridae*.

Despite the valuable information on Nile tilapia virome components and the divergent amnoonviruses roaming aquaculture facilities, this study, nonetheless, presents some limitations. The accuracy of the analysis could be improved in future studies on topics similar to this one with the newest version of ONT Flowcell used (R10). Although metagenomic sequencing offers lower coverage than targeted sequencing, it allows for an unbiased analysis of the samples and therefore for a broader overview of the sample composition [85]. Other technologies, such as Illumina, might offer shorter but numerically more reads (therefore with potentially higher coverage depth) [86]. However, the technology is not usually suitable for deployment at the point of need and is less cost-efficient for fewer samples. In this study, a simple and user-friendly approach was used through the rapid barcoding kit. This allowed for a first overview of the Nile tilapia virome richness and abundance.

## 5. Conclusions

The ONT sequencing platform can provide valuable information regarding the virome of Nile tilapia. Interestingly, 9 of the 10 components of the tilapia virome are not known to cause pathological conditions in fish; however, their exact identity needs to be confirmed in follow-up studies. The amnoonvirus detected in this study, though clustered with members of *Tilapinevirus*, was diverged enough to form a distinct lineage. Phylogenetic analyses performed on partial sequences of segments 1, 5, and 7 showed the robust phylogenetic distinction of AmnoonvirusEGY1 from other members of the genus *Tilapinevirus*, yet comparisons using full genome sequences are needed.

## Figures and Tables

**Table 2:** Virome components in Nile tilapia collected from five sampling sites.

	Husseiniya	Bahr Yusef	Ward Island	Om Khalaf	Lake Mariout
*Muvirus sfmu*					
*Muvirus mu*					
*Muvirus* 108					
*Gorganvirus isfahan*					
Unclassified *Podovirus*					
*Betabaculovirus chofumeferanae*					
*Shamonda orthobunyavirus*					
*Amnoonvirus* sp.					
*Pandoravirus* sp.					
*Mollivirus* sp.					

Blue cells indicate presence, white cells indicate absence.

**Table 3:** Percent of relative abundance (%) of virome components among different organs and sampling sites (Shaded cells: Not done).

Organ	Virus	Husseiniya (%)	Bahr Yusef (%)	Ward Island (%)	Om Khalaf (%)	Lake Mariout (%)
Gills	*Muvirus sfmu*	16.4	70			
	*Muvirus mu*	11.9	30			
	*Amnoonvirus* sp.	66.3	0			
	*Betabaculovirus chofumeferanae*	0.1	0			
	*Shamonda orthobunyavirus*	5.3	0			
Brain	*Muvirus sfmu*	44.8	26.1	46.7	63	67.5
	*Muvirus mu*	36.6	15	17.7	37	30
	*Amnoonvirus* sp.	7.7	31.3	1.3	0	0.1
	*Betabaculovirus chofumeferanae*	0.4	3	33.6	0	2.4
	*Shamonda orthobunyavirus*	9.8	24	0	0	0
	*Gorganvirus isfahan*	0.7	0.6	0	0	0
	*Muvirus* 108	0	0	0.7	0	0
Liver	*Muvirus sfmu*	64	32.5	66.4	55	80
	*Muvirus mu*	25.6	67.5	33.6	45	20
	*Amnoonvirus* sp.	1.9	0	0	0	0
	*Betabaculovirus chofumeferanae*	0.2	0	0	0	0
	*Shamonda orthobunyavirus*	8.3	0	0	0	0
Spleen	*Muvirus sfmu*	45.65	66.5	33	67	43
	*Muvirus mu*	54.3	33.5	45	33	57
	*Betabaculovirus chofumeferanae*	0	0	0.6	0	0
	*Shamonda orthobunyavirus*	0	0	21.4	0	0
	*Mollivirus* sp.	0.04	0	0	0	0
	*Pandoravirus* sp.	0.01	0	0	0	0
Kidney	*Muvirus sfmu*	37.7	58	27.4		
	*Muvirus mu*	48	13.3	55.6		
	*Amnoonvirus* sp.	11.2	10	0		
	*Betabaculovirus chofumeferanae*	0.3	0.3	17		
	*Shamonda orthobunyavirus*	2.4	17.4	0		
	Unclassified *Podovirus*	0.4	0	0		
	*Muvirus* 108	0	1	0		

**Table 4:** Viral species richness in Nile tilapia per organ and sampling site.

	Husseiniya	Bahr Yusef	Ward island	Om Khalaf	Lake Mariout	Total
Gills	5	2	0	0	0	5
Liver	5	2	2	2	2	5
Spleen	4	2	4	2	2	6
Kidneys	6	6	3	0	0	7
Brain	6	6	5	2	4	7
Total	9	7	5	2	4	

## Data Availability

Partial sequences of segments 1, 5, and 7 of AmnoonvirusEGY1 are deposited at (https://zenodo.org/records/15733869, accessed on 26 June 2025).

## Supplementary Materials

The following supporting information can be downloaded at https://www.mdpi.com/article/10.3390/pathogens14090935/s1, Table S1: Number of Nile tilapia collected from five sampling sites in Egypt, tissue type, and number of pooled samples collected for metagenomic analysis following RNA and/or DNA extraction. Table S2: Pairwise nucleotide sequence distances based on the PB1 gene between AmnoonvirusEGY1 isolate (of this study) and representative members of the order Articulavirales. Distances were calculated using the *p*-distance model and are expressed as the percentage of differing nucleotide sites between sequences. Lower values indicate higher sequence similarity. Table S3: Pairwise amino acid sequences distances based on the PB1 gene between the Egyptian Amnoonvirus isolate (this study) and representative members of the order Articulavirales. Distances were calculated using the *p*-distance model and are expressed as the percentage of differing amino acid sites between sequences. Lower values indicate higher sequence similarity. Table S4: Pairwise nucleotide sequences distances based on the PB1 gene between AmnoonvirusEGY1 isolate (of this study) and representative members of the *Amnoonviridae* family (classified, and unclassified). Distances were calculated using the *p*-distance model and are expressed as the percentage of differing nucleotide sites between sequences. Lower values indicate higher sequence similarity. Table S5: Pairwise amino acid sequences distances based on the PB1 gene between AmnoonvirusEGY1 isolate (this study) and representative members of the *Amnoonviridae* family (classified, and unclassified). Distances were calculated using the *p*-distance model and are expressed as the percentage of differing amino acid sites between sequences. Lower values indicate higher sequence similarity. Table S6: Pairwise nucleotide sequence distances between AmnoonvirusEGY1 isolate (this study) and different *Amnoonviridae* members, including the TiLV, TiLV-like, and FTGV by segment 5. Distances were calculated using the p-distance model and are expressed as the percentage of differing nucleotide sites between sequences. Lower values indicate higher sequence similarity. Table S7: Pairwise amino acid sequence distances between AmnoonvirusEGY1 isolate (this study) and different *Amnoonviridae* members, including the TiLV, TiLV-like, and FTGV by segment 5. Distances were calculated using the *p*-distance model and are expressed as the percentage of differing amino acid sites between sequences. Lower values indicate higher sequence similarity. Table S8: Pairwise nucleotide sequence distances between AmnoonvirusEGY1 isolate (this study) and different *Amnoonviridae* members, including the TiLV and TiLV-like by segment 7. Distances were calculated using the *p*-distance model and are expressed as the percentage of differing nucleotide sites between sequences. Lower values indicate higher sequence similarity. Table S9: Pairwise amino acid sequence distances between AmnoonvirusEGY1 isolate (this study) and different *Amnoonviridae* members, including the TiLV and TiLV-like by segment 7. Distances were calculated using the *p*-distance model and are expressed as the percentage of differing amino acid sites between sequences. Lower values indicate higher sequence similarity. Figure S1. An unrooted maximum likelihood phylograms illustrating the phylogenetic relationships between AmnoonvirusEGY1 nucleotide sequences (indicated by red ★) and TiLV strains from Israel for genomic segments 1, 5, and 7. A. Segment 1 was aligned with four Israeli strains (GenBank Accession Nos. OP037908, OP037898, KU552131, and KU751814). B. Segment 5 was compared with three Israeli strains (OP037912, OP037902, and KU751818). C. Segment 7 was analyzed against four Israeli strains (OP037914, OP037904, KU552137, and KU751820). Each viral isolate is labeled with its GenBank accession number, virus name, country of origin, and isolate designation. Bootstrap values ≥ 70% are displayed at corresponding nodes to indicate clade support. Scale bars represent the number of nucleotide substitutions per site: 0.050 for panel A, 0.20 for panel B, and 0.10 for panel C.
