# Supplementary material for: Exploring the Virome of Nile Tilapia (Oreochromis niloticus) Using Metagenomic Analysis
Source: Pathogens. 2025 Sep 16;14(9):935. doi: 10.3390/pathogens14090935 (PMC12472937; doi:10.3390/pathogens14090935)
Supplement: Supplementary file 1 [file pathogens-14-00935-s001.zip › pathogens-3755190-supplementary.pdf]

Table S1: Number of Nile tilapia collected from five sampling sites in Egypt, tissue type, and number of pooled samples collected for metagenomic analysis following RNA and/or DNA extraction

Sampling site	Liver	Kidney	Spleen	Brain	Gills
Husseiniya	5 fish*<br>RNA=2**<br>DNA=2***	5 fish<br>RNA=2<br>DNA=2	5 fish<br>RNA=2<br>DNA=1	5 fish<br>RNA=2<br>DNA=2	5 fish<br>RNA=2<br>DNA=2
Mariout	5 fish<br>RNA=2	ND****	4 fish<br>RNA=1	5 fish<br>RNA=1<br>DNA=2	ND
Ward Island	3 fish<br>RNA=1<br>DNA=1	3 fish<br>RNA=1<br>DNA=1	3 fish<br>RNA=1<br>DNA=1	3 fish<br>RNA=1	ND
Om Khalaf	3 fish<br>RNA=1	ND	3 fish<br>RNA=1	3 fish<br>RNA=2	ND
Bahr Yusef	2 fish<br>RNA=1<br>DNA=1	2 fish<br>RNA=1<br>DNA=1	2 fish<br>RNA=1<br>DNA=1	2 fish<br>RNA=2	2 fish<br>DNA=2
Total	18 fish<br>RNA=7<br>DNA=4	10 fish<br>RNA=4<br>DNA=4	17 fish<br>RNA=6<br>DNA=3	18 fish RNA=8<br>DNA=4	7 fish<br>RNA=2<br>DNA=4

*Number of fish sampled
**Number of pooled samples extracted for RNA
***Number of pooled samples extracted for DNA
****Not done

Table S2: Pairwise nucleotide sequence distances based on the PB1 gene between AmnoonvirusEGY1 isolate (of this study) and representative members of the order Articulavirales. Distances were calculated using the *p*-distance model and are expressed as the percentage of differing nucleotide sites between sequences. Lower values indicate higher sequence similarity.

	AmnoonvirusEGY1	TiLV*_Index_strain	Rainbowtrout_orthomyxovirus1	Infectious_Salmon_Anemia_virus	Yancheng_orthomyxo-like_virus	Wenling_orthomyxo-like_virus2	Wenling_hagfish_influenza_virus	Wuhan_asiatic_toad_influenza_virus	Wuhan_spiny_eel_influenza_virus	Wuhan_carp_Isavirus1	Xibalbanus_thogotovirus1	Influenza_A	Influenza_B	Influenza_C	Influenza_D	Pilchard_orthomyxovirus	Lautavirus	TiLV_like_virus1	Fancy_Tailed_Guppy_Virus_B2	Flavolineata_virus	Dolomieu_virus	Namensis_virus	Stewartii_virus	Przewalskiivirus	Asotusvirus1	Asotusvirus2
**AmnoonvirusEGY1**																										
**TiLV*_Index_strain**	13.38																									
**Rainbowtrout_orthomyxovirus1**	74.23	74.49																								
**Infectious_Salmon_Anemia_virus**	75.51	75.26	45.66																							
**Yancheng_orthomyxo-like_virus**	76.55	76.03	73.45	69.07																						
**Wenling_orthomyxo-like_virus2**	73.71	73.45	68.04	70.62	71.39																					
**Wenling_hagfish_influenza_virus**	68.70	68.19	73.98	74.49	76.80	69.33																				
**Wuhan_asiatic_toad_influenza_virus**	66.92	64.90	69.90	71.17	76.29	71.91	55.98																			
**Wuhan_spiny_eel_influenza_virus**	75.70	76.96	66.07	62.76	73.20	71.65	73.79	66.33																		
**Wuhan_carp_Isavirus1**	70.92	72.45	48.21	44.39	71.65	70.36	67.86	70.66	63.27																	
**Xibalbanus_thogotovirus1**	73.82	74.35	71.99	73.56	79.32	70.16	68.06	71.20	68.59	70.68																
**Influenza_A**	75.44	76.71	61.73	59.44	71.91	68.56	68.70	67.09	42.28	57.91	69.63															
**Influenza_B**	74.43	74.43	61.48	58.16	72.68	69.59	71.50	66.58	33.42	59.44	67.80	40.00														
**Influenza_C**	73.16	74.18	62.50	65.82	65.21	69.85	67.43	63.80	50.13	61.22	65.97	46.08	47.34													
**Influenza_D**	75.44	75.70	61.22	60.20	70.36	67.78	68.45	62.53	52.66	58.16	67.54	48.86	48.10	34.18												
**Pilchard_orthomyxovirus**	73.21	73.72	47.70	48.21	70.88	69.07	73.47	71.43	66.84	46.94	77.75	61.99	65.31	63.78	62.76											
**Lautavirus**	69.21	69.21	76.79	76.79	72.68	73.20	73.03	73.28	75.57	75.00	75.92	75.06	75.57	74.30	74.30	75.26										
**TiLV_like_virus1**	23.48	17.42	74.23	72.96	75.26	76.03	66.92	61.87	75.19	70.92	74.08	75.70	73.16	72.66	75.19	75.00	68.96									
**Fancy_Tailed_Guppy_Virus_B2**	22.47	17.68	73.72	73.47	75.52	76.03	67.43	63.64	75.70	70.15	74.35	76.20	74.18	71.65	74.43	74.23	68.96	7.58								
**Flavolineata_virus**	58.88	59.39	74.49	73.98	72.16	78.09	65.90	68.53	67.77	70.66	74.61	71.57	70.81	70.81	73.35	72.45	70.99	59.14	58.63							
**Dolomieu_virus**	78.32	80.06	76.59	73.70	73.99	78.90	76.30	74.57	71.39	72.83	76.59	74.28	71.39	77.46	76.88	75.72	74.57	79.77	79.19	77.17						
**Namensis_virus**	59.54	60.05	73.98	72.45	72.16	74.23	67.68	68.70	77.35	71.43	75.13	72.77	72.77	71.50	72.01	74.23	71.50	56.74	57.51	58.52	79.48					
**Stewartii_virus**	70.95	71.72	78.15	77.89	75.26	76.55	77.12	76.09	75.32	75.32	74.87	74.04	75.84	73.52	75.58	78.15	73.78	72.24	73.26	70.18	75.14	77.12				
**Przewalskiivirus**	75.51	76.02	69.13	69.39	69.33	75.52	70.66	73.98	67.60	69.13	72.51	71.17	69.64	70.92	69.13	66.07	76.53	74.74	76.02	72.70	76.01	73.47	73.01			
**Asotusvirus1**	72.45	73.98	70.92	71.17	76.55	75.52	75.77	73.98	76.28	72.45	78.27	72.45	74.49	75.00	73.21	69.64	71.68	75.26	73.98	72.70	71.68	71.94	75.58	70.66		
**Asotusvirus2**	69.95	71.24	74.35	73.32	70.21	75.91	77.72	73.58	74.61	75.65	77.75	71.50	73.83	74.87	76.17	74.87	70.21	70.73	70.47	73.32	76.88	73.32	73.32	75.39	71.50	

Table S3: Pairwise amino acid sequences distances based on the PB1 gene between the Egyptian Amnoonvirus isolate (this study) and representative members of the order Articulavirales. Distances were calculated using the *p*-distance model and are expressed as the percentage of differing amino acid sites between sequences. Lower values indicate higher sequence similarity.

	AmnoonvirusEGY1	TiLV*_Index_strain	Rainbowtrout_orthomyxovirus1	Infectious_Salmon_Anemia_virus	Yancheng_orthomyxo-like_virus	Wenling_orthomyxo-like_virus2	Wenling_hagfish_influenza_virus	Wuhan_asiatic_toad_influenza_virus	Wuhan_spiny_eel_influenza_virus	Wuhan_carp_Isavirus1	Xibalbanus_thogotovirus1	Influenza_A	Influenza_B	Influenza_C	Influenza_D	Pilchard_orthomyxovirus	Lautavirus	TiLV_like_virus1	Fancy_Tailed_Guppy_Virus_B2	Flavolineata_virus	Dolomieu_virus	Namensis_virus	Stewartii_virus	Przewalskiivirus	Asotusvirus1	Asotusvirus2
**AmnoonvirusEGY1**																										
**TiLV*_Index_strain**	16.33																									
**Rainbowtrout_orthomyxovirus1**	95.83	95.83																								
**Infectious_Salmon_Anemia_virus**	97.92	97.92	70.83																							
**Yancheng_orthomyxo-like_virus**	95.83	97.92	89.58	91.67																						
**Wenling_orthomyxo-like_virus2**	95.83	95.83	91.67	91.67	93.75																					
**Wenling_hagfish_influenza_virus**	93.75	93.75	89.58	93.75	100	85.42																				
**Wuhan_asiatic_toad_influenza_virus**	95.92	95.92	91.67	97.92	95.83	89.58	85.42																			
**Wuhan_spiny_eel_influenza_virus**	97.92	97.92	91.67	91.67	91.67	95.83	93.75	87.50																		
**Wuhan_carp_Isavirus1**	93.75	95.83	72.92	60.42	89.58	95.83	85.42	91.67	85.42																	
**Xibalbanus_thogotovirus1**	89.58	91.67	93.75	97.92	95.83	95.83	87.50	91.67	87.50	97.92																
**Influenza_A**	91.67	93.75	91.67	87.50	91.67	89.58	91.67	89.58	58.33	89.58	91.67															
**Influenza_B**	89.58	93.75	81.25	83.33	83.33	91.67	89.58	85.42	54.17	87.50	85.42	64.58														
**Influenza_C**	87.50	89.58	93.75	93.75	87.50	95.83	89.58	77.08	77.08	91.67	87.50	70.83	72.92													
**Influenza_D**	89.58	89.58	85.42	87.50	85.42	93.75	93.75	89.58	77.08	83.33	91.67	66.67	66.67	52.08												
**Pilchard_orthomyxovirus**	97.92	97.92	66.67	68.75	93.75	95.83	93.75	89.58	91.67	68.75	93.75	91.67	85.42	93.75	89.58											
**Lautavirus**	91.67	89.58	93.75	89.58	89.58	95.83	93.75	95.83	91.67	87.50	93.75	87.50	95.83	89.58	85.42	93.75										
**TiLV_like_virus1**	44.90	42.86	93.75	93.75	97.92	95.83	95.83	93.88	97.92	89.58	85.42	93.75	97.92	89.58	91.67	95.83	89.58									
**Fancy_Tailed_Guppy_Virus_B2**	40.82	38.78	93.75	93.75	100	100	97.92	95.92	95.83	89.58	87.50	91.67	97.92	89.58	91.67	95.83	91.67	22.45								
**Flavolineata_virus**	77.08	75.00	89.58	93.75	95.83	93.75	87.50	95.83	93.75	95.83	95.83	97.92	89.58	89.58	93.75	93.75	93.75	81.25	75.00							
**Dolomieu_virus**	97.67	97.67	90.70	90.70	100	95.35	93.02	95.35	93.02	90.70	97.67	93.02	95.35	95.35	95.35	93.02	90.70	97.67	95.35	93.02						
**Namensis_virus**	85.42	85.42	89.58	91.67	93.75	89.58	93.75	85.42	91.67	93.75	95.83	85.42	85.42	89.58	85.42	91.67	95.83	79.17	81.25	89.58	100					
**Stewartii_virus**	87.50	93.75	95.83	95.83	87.50	93.75	95.83	93.75	93.75	93.75	93.75	89.58	95.83	89.58	91.67	97.92	91.67	91.67	95.83	91.67	93.02	93.75				
**Przewalskiivirus**	95.83	97.92	95.83	97.92	91.67	95.83	93.75	93.75	87.50	91.67	89.58	95.83	91.67	95.83	93.75	91.67	93.75	91.67	91.67	95.83	100.00	91.67	87.50			
**Asotusvirus1**	95.83	95.83	91.67	93.75	95.83	93.75	91.67	91.67	97.92	93.75	95.83	89.58	100	97.92	95.83	95.83	91.67	93.75	93.75	93.75	95.35	95.83	87.50	87.50		
**Asotusvirus2**	91.67	95.83	89.58	91.67	91.67	93.75	97.92	81.25	97.92	89.58	89.58	93.75	91.67	89.58	91.67	91.67	91.67	91.67	95.83	97.92	97.67	87.50	93.75	91.67	91.67	

Table S4: Pairwise nucleotide sequences distances based on the PB1 gene between AmnoonvirusEGY1 isolate (of this study) and representative members of the *Amnoonviridae* family (classified, and unclassified). Distances were calculated using the *p*-distance model and are expressed as the percentage of differing nucleotide sites between sequences. Lower values indicate higher sequence similarity.

	**AmnoonvirusEGY1**	**TilapiaLakeVirus**	**Flavolineatavirus**	**Dolomieuvirus**	**Namensisvirus**	**Stewartiivirus**	**Plagiostomusvirus**	**Przewalskiivirus**	**Asotusvirus1**	**Asotusvirus2**	**Fancy-TailedGuppyVirus**	**TilapiaLakeVirus-LikeVirus**
**AmnoonvirusEGY1**												
**TilapiaLakeVirus**	**13.13**											
**Flavolineatavirus**	**61.32**	**59.03**										
**Dolomieuvirus**	**69.11**	**68.29**	**60.83**									
**Namensisvirus**	**58.78**	**57.76**	**58.52**	**69.17**								
**Stewartiivirus**	**70.98**	**72.41**	**77.10**	**73.15**	**72.17**							
**Plagiostomusvirus**	**71.21**	**71.21**	**62.12**	**66.67**	**69.70**	**71.21**						
**Przewalskiivirus**	**68.17**	**70.27**	**70.91**	**73.17**	**67.58**	**74.25**	**69.70**					
**Asotusvirus1**	**71.22**	**68.42**	**76.11**	**78.86**	**77.29**	**71.61**	**74.60**	**75.65**				
**Asotusvirus2**	**71.30**	**69.28**	**69.01**	**73.17**	**67.54**	**72.06**	**69.70**	**69.97**	**69.88**			
**Fancy-TailedGuppyVirus**	**22.47**	**18.43**	**56.74**	**63.41**	**57.00**	**72.41**	**71.21**	**70.87**	**70.76**	**71.59**		
**TilapiaLakeVirus-LikeVirus**	**23.23**	**17.42**	**58.27**	**65.04**	**55.73**	**70.98**	**71.21**	**71.47**	**71.64**	**73.91**	**8.08**	

Table S5: Pairwise amino acid sequences distances based on the PB1 gene between AmnoonvirusEGY1 isolate (this study) and representative members of the *Amnoonviridae* family (classified, and unclassified). Distances were calculated using the *p*-distance model and are expressed as the percentage of differing amino acid sites between sequences. Lower values indicate higher sequence similarity.

	AmnoonvirusEGY1	TilapiaLakeVirus	Flavolineatavirus	Dolomieuvirus	Namensisvirus	Stewartiivirus	Plagiostomusvirus	Przewalskiivirus	Asotusvirus1	Asotusvirus2	Fancy-TailedGuppyVirus	TilapiaLakeVirus-LikeVirus
**AmnoonvirusEGY1**												
**TilapiaLakeVirus**	**19.19**											
**Flavolineatavirus**	**87.76**	**82.65**										
**Dolomieuvirus**	**92.86**	**89.29**	**81.48**									
**Namensisvirus**	**84.69**	**82.65**	**81.63**	**92.59**								
**Stewartiivirus**	**87.06**	**85.88**	**96.43**	**95.83**	**90.48**							
**Plagiostomusvirus**	**82.35**	**76.47**	**64.71**	**100.00**	**88.24**	**94.12**						
**Przewalskiivirus**	**86.08**	**86.08**	**88.46**	**85.71**	**80.77**	**94.29**	**94.12**					
**Asotusvirus1**	**91.67**	**88.10**	**95.18**	**89.29**	**92.77**	**82.05**	**93.75**	**84.93**				
**Asotusvirus2**	**82.14**	**80.95**	**87.95**	**89.29**	**84.34**	**92.11**	**88.24**	**83.10**	**84.52**			
**Fancy-TailedGuppyVirus**	**41.41**	**36.36**	**80.61**	**82.14**	**82.65**	**89.41**	**76.47**	**88.61**	**91.67**	**89.29**		
**TilapiaLakeVirus-LikeVirus**	**40.40**	**35.35**	**85.71**	**85.71**	**81.63**	**89.41**	**76.47**	**88.61**	**94.05**	**89.29**	**14.14**	

Table S6: Pairwise nucleotide sequence distances between AmnoonvirusEGY1 isolate (this study) and different *Amnoonviridae* members, including the TiLV, TiLV-like, and FTGV by segment 5. Distances were calculated using the p-distance model and are expressed as the percentage of differing nucleotide sites between sequences. Lower values indicate higher sequence similarity.

	AmnoonvirusEGY1	OP037912/TiLV/Israel/939-16/2018	MK392376/TiLV/Ecuador/EC-2012	OP037902/TiLV/Israel/939-9/2018	MT466441/TiLV/Bangladesh/BD-2017-181	MT466451/TiLV/Bangladesh/BD-2019-E1	MN687769/TiLV/Thailand/TH-2019	MN687729/TiLV/Thailand/TH-2016-CN	MN687689/TiLV/Thailand/TH-2013	MN193517/TiLV/USA/WVL19031-01A	MN687719/TiLV/Thailand/TH-2016-CU	OM469313/TiLV/India/M25/SRLAAH/2021	MN193527/TiLV/USA/WVL19054	MN687749/TiLV/Thailand/TH-2018-N	MH319382/TiLV/Thailand/WVL18053-01A	MT466461/TiLV/Bangladesh/BD-2019-E3	OR101703/TiLV/India/KR-5/S2/SRLAAH/2021	MN687709/TiLV/Thailand/TH-2015	ON376586/TiLV/Viet_Nam/RIA2-VN-2019	MN687759/TiLV/Thailand/TH-2018-K	MK425014/TiLV/Peru/F3-4	OR101704/TiLV/India/CC-5/S4/SRLAAH/2023	OL469277/TiLV/Thailand/TiLV-2018	MN687739/TiLV/Thailand/TH-2017	KX631925/TiLV/Thailand/TV1	MZ297927/TiLV/India/IND-2018	KU751818/TiLV/Israel/Til-4-2011*Index_strian	BK063204/TiLV-like_virus/Caribbean/Maracas_2015-1	BK063214/TiLV-like_virus/Caribbean/Maracas_2015-2	PP409989/FTGV/USA/Guppy/95/10/82	PP409999/FTGV/USA/CobraB-2	MN939376/TiLV/Bangladesh/BD-2017	OQ437058/TiLV/Hong_Kong/Israel-HK	KU552141/TilapiaVirus/Israrl/AD-2016	KY615743/TiLV/Thailand/CL	PV356101/TiLV/Colombia/ICA2023	ON376576/TiLV/Viet_Nam/HB196-VN-2020
AmnoonvirusEGY1																																					
OP037912/TiLV/Israel/939-16/2018	60.98																																				
MK392376/TiLV/Ecuador/EC-2012	59.95	3.62																																			
OP037902/TiLV/Israel/939-9/2018	60.47	1.55	3.62																																		
MT466441/TiLV/Bangladesh/BD-2017-181	59.43	4.91	2.33	4.91																																	
MT466451/TiLV/Bangladesh/BD-2019-E1	59.69	4.91	2.33	4.91	0.52																																
MN687769/TiLV/Thailand/TH-2019	60.21	2.58	2.07	2.58	3.36	3.36																															
MN687729/TiLV/Thailand/TH-2016-CN	60.47	2.33	1.81	2.33	3.10	3.10	0.78																														
MN687689/TiLV/Thailand/TH-2013	59.95	4.39	1.81	4.39	3.62	3.62	2.84	2.58																													
MN193517/TiLV/USA/WVL19031-01A	60.21	2.07	2.58	2.07	3.88	3.88	1.03	1.29	3.36																												
MN687719/TiLV/Thailand/TH-2016-CU	59.95	3.36	1.81	3.36	3.10	3.10	1.81	1.55	3.10	2.33																											
OM469313/TiLV/India/M25/SRLAAH/2021	59.17	5.43	2.84	5.43	0.52	1.03	3.88	3.62	4.13	4.39	3.10																										
MN193527/TiLV/USA/WVL19054	60.21	2.58	3.10	2.58	4.39	4.39	1.55	1.81	3.88	0.52	2.84	4.91																									
MN687749/TiLV/Thailand/TH-2018-N	60.72	3.10	3.10	3.10	4.39	4.39	1.55	1.81	3.88	1.55	2.84	4.91	2.07																								
MH319382/TiLV/Thailand/WVL18053-01A	59.95	3.36	1.81	3.36	3.10	3.10	1.81	1.55	3.10	2.33	0.00	3.10	2.84	2.84																							
MT466461/TiLV/Bangladesh/BD-2019-E3	59.95	5.43	2.84	5.43	0.52	0.52	3.88	3.62	4.13	4.39	3.62	1.03	4.91	4.65	3.62																						
OR101703/TiLV/India/KR-5/S2/SRLAAH/2021	59.43	5.43	3.10	5.68	0.78	1.29	4.13	3.88	4.39	4.65	3.36	0.26	5.17	5.17	3.36	1.29																					
MN687709/TiLV/Thailand/TH-2015	60.21	4.91	2.33	4.91	3.10	3.10	3.36	3.10	3.62	3.88	3.10	3.62	4.39	3.88	3.10	3.62	3.88																				
ON376586/TiLV/Viet_Nam/RIA2-VN-2019	60.94	9.38	7.55	8.33	7.81	7.81	7.81	7.55	8.85	8.33	8.07	8.33	8.85	8.85	8.07	8.33	8.59	7.29																			
MN687759/TiLV/Thailand/TH-2018-K	60.98	5.43	2.33	5.43	3.62	4.13	3.88	3.62	4.13	4.39	3.62	4.13	4.91	4.39	3.62	4.13	4.39	3.62	8.33																		
MK425014/TiLV/Peru/F3-4	60.21	4.65	1.03	4.65	3.36	3.36	3.10	2.84	2.84	3.62	2.84	3.88	4.13	4.13	2.84	3.88	4.13	3.36	8.07	2.84																	
OR101704/TiLV/India/CC-5/S4/SRLAAH/2023	60.47	6.20	4.13	6.20	5.94	5.94	4.65	4.91	3.36	5.17	5.43	6.46	5.68	5.68	5.43	6.46	6.46	5.43	9.64	6.46	4.65																
OL469277/TiLV/Thailand/TiLV-2018	59.69	4.39	2.84	4.39	3.62	3.62	2.84	2.58	4.13	3.36	1.03	3.62	3.88	3.88	1.03	4.13	3.88	4.13	8.59	4.65	3.88	6.46															
MN687739/TiLV/Thailand/TH-2017	60.21	5.17	3.10	5.17	3.88	3.88	4.13	3.88	4.39	4.65	3.88	4.39	5.17	4.65	3.88	4.39	4.65	1.29	7.81	4.13	3.62	6.20	4.91														
KX631925/TiLV/Thailand/TV1	59.69	6.20	3.62	6.20	4.39	4.39	4.65	4.39	4.91	5.17	3.88	4.91	5.68	5.17	3.88	4.91	5.17	3.36	8.07	4.91	4.65	6.20	4.91	4.65													
MZ297927/TiLV/India/IND-2018	60.05	6.88	3.70	5.82	5.56	5.56	5.29	5.03	5.03	5.56	5.56	6.08	6.08	6.35	5.56	6.08	6.35	5.56	9.60	6.08	4.76	7.41	6.61	6.35	6.35												
KU751818/TiLV/Israel/Til-4-2011*Index_strian	59.95	3.62	0.00	3.62	2.33	2.33	2.07	1.81	1.81	2.58	1.81	2.84	3.10	3.10	1.81	2.84	3.10	2.33	7.55	2.33	1.03	4.13	2.84	3.10	3.62	3.70											
BK063204/TiLV-like_virus/Caribbean/Maracas_2015-1	61.46	19.53	19.01	19.53	20.57	20.57	18.49	18.75	20.05	17.97	19.27	20.57	17.97	17.71	19.27	20.57	20.57	18.49	18.37	19.53	19.01	20.83	20.31	19.79	20.57	20.00	19.01										
BK063214/TiLV-like_virus/Caribbean/Maracas_2015-2	61.46	19.53	19.01	19.53	20.57	20.57	18.49	18.75	20.05	17.97	19.27	20.57	17.97	17.71	19.27	20.57	20.57	18.49	18.37	19.53	19.01	20.83	20.31	19.79	20.57	20.00	19.01	0.00									
PP409989/FTGV/USA/Guppy/95/10/82	61.98	19.53	18.23	19.01	20.05	20.05	17.45	18.23	19.27	17.45	18.75	20.05	17.45	17.19	18.75	20.05	20.31	18.49	18.11	18.75	18.75	20.05	19.27	18.23	20.05	19.73	18.23	8.07	8.07								
PP409999/FTGV/USA/CobraB-2	60.94	18.49	18.49	18.49	20.31	19.79	17.97	18.75	19.53	17.97	19.27	20.31	17.97	17.71	19.27	19.79	20.31	18.75	18.37	18.49	19.01	20.31	20.31	17.97	20.83	20.00	18.49	8.33	8.33	6.77							
MN939376/TiLV/Bangladesh/BD-2017	59.43	5.43	2.84	5.43	0.52	1.03	3.88	3.62	4.13	4.39	3.62	1.03	4.91	4.91	3.62	1.03	1.29	3.62	7.81	4.13	3.36	6.46	4.13	3.88	4.91	6.08	2.84	20.83	20.83	20.31	20.57						
OQ437058/TiLV/Hong_Kong/Israel-HK	59.59	4.15	1.04	4.15	2.85	2.85	2.59	2.33	2.33	3.11	2.33	3.37	3.63	3.37	2.33	3.37	3.63	2.59	7.57	3.11	2.07	4.15	3.37	3.37	3.37	4.77	1.04	19.84	19.84	19.06	19.32	3.37					
KU552141/TilapiaVirus/Israrl/AD-2016	60.21	3.62	1.55	4.13	3.36	3.36	3.10	2.84	2.84	3.62	2.84	3.88	4.13	4.13	2.84	3.88	3.88	2.84	7.81	3.88	2.58	4.65	3.88	3.62	4.65	5.29	1.55	19.27	19.27	19.01	18.23	3.88	2.07				
KY615743/TiLV/Thailand/CL	59.95	3.10	1.55	3.10	2.84	2.84	1.55	1.29	2.84	2.07	0.26	2.84	2.58	2.58	0.26	3.36	3.10	2.84	7.81	3.36	2.58	5.17	1.29	3.62	3.62	5.29	1.55	19.01	19.01	18.49	19.01	3.36	2.07	2.58			
PV356101/TiLV/Colombia/ICA2023	60.72	5.17	3.10	5.68	4.91	4.91	4.13	3.88	4.91	4.65	3.88	5.43	5.17	5.17	3.88	5.43	5.68	4.91	8.59	4.39	4.13	7.24	4.91	5.68	5.68	6.88	3.10	20.05	20.05	18.49	19.27	5.43	4.15	4.65	3.62		
ON376576/TiLV/Viet_Nam/HB196-VN-2020	60.21	3.88	4.91	3.36	6.20	6.20	3.36	3.62	5.68	2.84	4.13	6.72	2.84	3.88	4.13	6.72	6.98	6.20	10.16	6.72	5.94	7.49	5.17	6.46	6.46	6.88	4.91	19.01	19.01	18.49	19.01	6.72	5.44	5.94	3.88	6.46	

Table S7: Pairwise amino acid sequence distances between AmnoonvirusEGY1 isolate (this study) and different *Amnoonviridae* members, including the TiLV, TiLV-like, and FTGV by segment 5. Distances were calculated using the *p*-distance model and are expressed as the percentage of differing amino acid sites between sequences. Lower values indicate higher sequence similarity.

.

	AmnoonvirusEGY1	OP037912/TiLV/Israel/939-16/2018	MK392376/TiLV/Ecuador/EC-2012	OP037902/TiLV/Israel/939-9/2018	MT466441/TiLV/Bangladesh/BD-2017-181	MT466451/TiLV/Bangladesh/BD-2019-E1	MN687769/TiLV/Thailand/TH-2019	MN687729/TiLV/Thailand/TH-2016-CN	MN687689/TiLV/Thailand/TH-2013	MN193517/TiLV/USA/WVL19031-01A	MN687719/TiLV/Thailand/TH-2016-CU	OM469313/TiLV/India/M25/SRLAAH/2021	MN193527/TiLV/USA/WVL19054	MN687749/TiLV/Thailand/TH-2018-N	MH319382/TiLV/Thailand/WVL18053-01A	MT466461/TiLV/Bangladesh/BD-2019-E3	OR101703/TiLV/India/KR-5/S2/SRLAAH/2021	MN687709/TiLV/Thailand/TH-2015	ON376586/TiLV/Viet_Nam/RIA2-VN-2019	MN687759/TiLV/Thailand/TH-2018-K	MK425014/TiLV/Peru/F3-4	OR101704/TiLV/India/CC-5/S4/SRLAAH/2023	OL469277/TiLV/Thailand/TiLV-2018	MN687739/TiLV/Thailand/TH-2017	KX631925/TiLV/Thailand/TV1	MZ297927/TiLV/India/IND-2018	KU751818/TiLV/Israel/Til-4-2011*Index_strian	BK063204/TiLV-like_virus/Caribbean/Maracas_2015-1	BK063214/TiLV-like_virus/Caribbean/Maracas_2015-2	PP409989/FTGV/USA/Guppy/95/10/82	PP409999/FTGV/USA/CobraB-2	MN939376/TiLV/Bangladesh/BD-2017	OQ437058/TiLV/Hong_Kong/Israel-HK	KU552141/TilapiaVirus/Israrl/AD-2016	KY615743/TiLV/Thailand/CL	PV356101/TiLV/Colombia/ICA2023	ON376576/TiLV/Viet_Nam/HB196-VN-2020
AmnoonvirusEGY1																																					
OP037912/TiLV/Israel/939-16/2018	76.67																																				
MK392376/TiLV/Ecuador/EC-2012	76.67	2.50																																			
OP037902/TiLV/Israel/939-9/2018	76.67	0.00	2.50																																		
MT466441/TiLV/Bangladesh/BD-2017-181	76.67	1.67	0.83	1.67																																	
MT466451/TiLV/Bangladesh/BD-2019-E1	76.67	1.67	0.83	1.67	0.00																																
MN687769/TiLV/Thailand/TH-2019	75.83	0.83	3.33	0.83	2.50	2.50																															
MN687729/TiLV/Thailand/TH-2016-CN	76.67	0.83	3.33	0.83	2.50	2.50	1.67																														
MN687689/TiLV/Thailand/TH-2013	76.67	5.00	2.50	5.00	3.33	3.33	5.83	5.00																													
MN193517/TiLV/USA/WVL19031-01A	76.67	0.00	2.50	0.00	1.67	1.67	0.83	0.83	5.00																												
MN687719/TiLV/Thailand/TH-2016-CU	76.67	0.00	2.50	0.00	1.67	1.67	0.83	0.83	5.00	0.00																											
OM469313/TiLV/India/M25/SRLAAH/2021	76.67	1.67	0.83	1.67	0.00	0.00	2.50	2.50	3.33	1.67	1.67																										
MN193527/TiLV/USA/WVL19054	75.83	0.83	3.33	0.83	2.50	2.50	1.67	1.67	5.83	0.83	0.83	2.50																									
MN687749/TiLV/Thailand/TH-2018-N	76.67	1.67	4.17	1.67	3.33	3.33	2.50	2.50	6.67	1.67	1.67	3.33	2.50																								
MH319382/TiLV/Thailand/WVL18053-01A	76.67	0.00	2.50	0.00	1.67	1.67	0.83	0.83	5.00	0.00	0.00	1.67	0.83	1.67																							
MT466461/TiLV/Bangladesh/BD-2019-E3	76.67	1.67	0.83	1.67	0.00	0.00	2.50	2.50	3.33	1.67	1.67	0.00	2.50	3.33	1.67																						
OR101703/TiLV/India/KR-5/S2/SRLAAH/2021	76.67	1.67	0.83	1.67	0.00	0.00	2.50	2.50	3.33	1.67	1.67	0.00	2.50	3.33	1.67	0.00																					
MN687709/TiLV/Thailand/TH-2015	76.67	2.50	1.67	2.50	0.83	0.83	3.33	3.33	4.17	2.50	2.50	0.83	3.33	4.17	2.50	0.83	0.83																				
ON376586/TiLV/Viet_Nam/RIA2-VN-2019	77.31	3.36	3.36	3.36	2.52	2.52	4.20	4.20	5.88	3.36	3.36	2.52	4.20	5.04	3.36	2.52	2.52	3.36																			
MN687759/TiLV/Thailand/TH-2018-K	77.50	2.50	1.67	2.50	0.83	0.83	3.33	3.33	4.17	2.50	2.50	0.83	3.33	4.17	2.50	0.83	0.83	1.67	3.36																		
MK425014/TiLV/Peru/F3-4	76.67	2.50	0.00	2.50	0.83	0.83	3.33	3.33	2.50	2.50	2.50	0.83	3.33	4.17	2.50	0.83	0.83	1.67	3.36	1.67																	
OR101704/TiLV/India/CC-5/S4/SRLAAH/2023	76.67	4.17	1.67	4.17	2.50	2.50	5.00	4.17	0.83	4.17	4.17	2.50	5.00	5.83	4.17	2.50	2.50	3.33	5.04	3.33	1.67																
OL469277/TiLV/Thailand/TiLV-2018	75.83	1.67	4.17	1.67	3.33	3.33	2.50	2.50	6.67	1.67	1.67	3.33	2.50	3.33	1.67	3.33	3.33	4.17	5.04	4.17	4.17	5.83															
MN687739/TiLV/Thailand/TH-2017	76.67	2.50	1.67	2.50	0.83	0.83	3.33	3.33	4.17	2.50	2.50	0.83	3.33	4.17	2.50	0.83	0.83	0.00	3.36	1.67	1.67	3.33	4.17														
KX631925/TiLV/Thailand/TV1	77.50	2.50	1.67	2.50	0.83	0.83	3.33	3.33	4.17	2.50	2.50	0.83	3.33	4.17	2.50	0.83	0.83	1.67	3.36	1.67	1.67	3.33	4.17	1.67													
MZ297927/TiLV/India/IND-2018	76.07	2.56	0.00	2.56	0.85	0.85	3.42	3.42	2.56	2.56	2.56	0.85	3.42	4.27	2.56	0.85	0.85	1.71	3.45	1.71	0.00	1.71	4.27	1.71	1.71												
KU751818/TiLV/Israel/Til-4-2011*Index_strian	76.67	2.50	0.00	2.50	0.83	0.83	3.33	3.33	2.50	2.50	2.50	0.83	3.33	4.17	2.50	0.83	0.83	1.67	3.36	1.67	0.00	1.67	4.17	1.67	1.67	0.00											
BK063204/TiLV-like_virus/Caribbean/Maracas_2015-1	77.50	17.50	17.50	17.50	17.50	17.50	18.33	18.33	20.00	17.50	17.50	17.50	18.33	18.33	17.50	17.50	17.50	18.33	14.29	18.33	17.50	19.17	19.17	18.33	17.50	17.09	17.50										
BK063214/TiLV-like_virus/Caribbean/Maracas_2015-2	77.50	17.50	17.50	17.50	17.50	17.50	18.33	18.33	20.00	17.50	17.50	17.50	18.33	18.33	17.50	17.50	17.50	18.33	14.29	18.33	17.50	19.17	19.17	18.33	17.50	17.09	17.50	0.00									
PP409989/FTGV/USA/Guppy/95/10/82	76.67	20.83	20.83	20.83	20.83	20.83	20.00	21.67	23.33	20.83	20.83	20.83	21.67	21.67	20.83	20.83	20.83	21.67	17.65	21.67	20.83	22.50	20.83	21.67	20.83	20.51	20.83	10.83	10.83								
PP409999/FTGV/USA/CobraB-2	76.67	15.83	15.83	15.83	15.83	15.83	15.00	16.67	18.33	15.83	15.83	15.83	16.67	16.67	15.83	15.83	15.83	16.67	12.61	16.67	15.83	17.50	17.50	16.67	15.83	16.24	15.83	8.33	8.33	7.50							
MN939376/TiLV/Bangladesh/BD-2017	76.67	1.67	0.83	1.67	0.00	0.00	2.50	2.50	3.33	1.67	1.67	0.00	2.50	3.33	1.67	0.00	0.00	0.83	2.52	0.83	0.83	2.50	3.33	0.83	0.83	0.85	0.83	17.50	17.50	20.83	15.83						
OQ437058/TiLV/Hong_Kong/Israel-HK	77.31	3.36	0.84	3.36	1.68	1.68	4.20	4.20	3.36	3.36	3.36	1.68	4.20	5.04	3.36	1.68	1.68	2.52	4.24	2.52	0.84	2.52	5.04	2.52	2.52	0.86	0.84	18.49	18.49	21.85	16.81	1.68					
KU552141/TilapiaVirus/Israrl/AD-2016	76.67	2.50	0.00	2.50	0.83	0.83	3.33	3.33	2.50	2.50	2.50	0.83	3.33	4.17	2.50	0.83	0.83	1.67	3.36	1.67	0.00	1.67	4.17	1.67	1.67	0.00	0.00	17.50	17.50	20.83	15.83	0.83	0.84				
KY615743/TiLV/Thailand/CL	76.67	0.00	2.50	0.00	1.67	1.67	0.83	0.83	5.00	0.00	0.00	1.67	0.83	1.67	0.00	1.67	1.67	2.50	3.36	2.50	2.50	4.17	1.67	2.50	2.50	2.56	2.50	17.50	17.50	20.83	15.83	1.67	3.36	2.50			
PV356101/TiLV/Colombia/ICA2023	76.67	1.67	1.67	1.67	0.83	0.83	2.50	2.50	4.17	1.67	1.67	0.83	2.50	3.33	1.67	0.83	0.83	1.67	2.52	1.67	1.67	3.33	3.33	1.67	1.67	1.71	1.67	17.50	17.50	20.83	15.83	0.83	2.52	1.67	1.67		
ON376576/TiLV/Viet_Nam/HB196-VN-2020	76.67	1.67	4.17	1.67	3.33	3.33	2.50	2.50	6.67	1.67	1.67	3.33	2.50	3.33	1.67	3.33	3.33	4.17	5.04	4.17	4.17	5.83	3.33	4.17	4.17	3.42	4.17	17.50	17.50	20.83	15.83	3.33	5.04	4.17	1.67	3.33	

Table S8: Pairwise nucleotide sequence distances between AmnoonvirusEGY1 isolate (this study) and different *Amnoonviridae* members, including the TiLV and TiLV-like by segment 7. Distances were calculated using the *p*-distance model and are expressed as the percentage of differing nucleotide sites between sequences. Lower values indicate higher sequence similarity.

	**AmnoonvirusEGY1**	**KU751820/TiLV/Israel/Til-4-2011*Index_strian**	**OM469315/TiLV/India/S27/SRLAAH/2021**	**OR101705/TiLV/India/KR-7/S2/SRLAAH/2021**	**OR101707/TiLV/India/CC-7/S4/SRLAAH/2022**	**OR101706/TiLV/India/RV-7/S3/SRLAAH/2021**	**OQ437060/TiLV/Hong_Kong/Israel-HK**	**KU552137/TilapiaVirus/Israrl/AD-2016**	**ON376578/TiLV/Viet_Nam/HB196-VN-2020**	**PV356103/TiLV/Colombia/ICA2023**	**MN939378/TiLV/Bangladesh/BD-2017**	**BK063216/TiLV-like_virus/Caribbean/Maracas_2015-2**	**BK063206/TiLV-like_virus/Caribbean/Maracas_2015-1**
**AmnoonvirusEGY1**													
**KU751820/TiLV/Israel/Til-4-2011*Index_strian**	**53.26**												
**OM469315/TiLV/India/S27/SRLAAH/2021**	**55.17**	**4.71**											
**OR101705/TiLV/India/KR-7/S2/SRLAAH/2021**	**54.79**	**2.90**	**1.81**										
**OR101707/TiLV/India/CC-7/S4/SRLAAH/2022**	**54.02**	**4.35**	**1.81**	**3.62**									
**OR101706/TiLV/India/RV-7/S3/SRLAAH/2021**	**54.79**	**2.90**	**4.71**	**2.90**	**3.62**								
**OQ437060/TiLV/Hong_Kong/Israel-HK**	**53.26**	**0.00**	**4.71**	**2.90**	**4.35**	**2.90**							
**KU552137/TilapiaVirus/Israrl/AD-2016**	**53.26**	**2.54**	**3.62**	**2.54**	**3.26**	**2.54**	**2.54**						
**ON376578/TiLV/Viet_Nam/HB196-VN-2020**	**52.11**	**5.43**	**6.16**	**5.43**	**5.80**	**5.43**	**5.43**	**4.35**					
**PV356103/TiLV/Colombia/ICA2023**	**54.02**	**3.62**	**5.07**	**3.62**	**4.71**	**2.90**	**3.62**	**2.54**	**4.71**				
**MN939378/TiLV/Bangladesh/BD-2017**	**54.79**	**4.35**	**1.09**	**2.90**	**1.45**	**4.35**	**4.35**	**3.26**	**5.80**	**4.71**			
**BK063216/TiLV-like_virus/Caribbean/Maracas_2015-2**	**52.87**	**19.05**	**20.15**	**19.78**	**19.78**	**19.78**	**19.05**	**19.41**	**19.78**	**19.41**	**19.78**		
**BK063206/TiLV-like_virus/Caribbean/Maracas_2015-1**	**52.87**	**19.05**	**20.15**	**19.78**	**19.78**	**19.78**	**19.05**	**19.41**	**19.78**	**19.41**	**19.78**	**0.00**	

Table S9: Pairwise amino acid sequence distances between AmnoonvirusEGY1 isolate (this study) and different *Amnoonviridae* members, including the TiLV and TiLV-like by segment 7. Distances were calculated using the *p*-distance model and are expressed as the percentage of differing amino acid sites between sequences. Lower values indicate higher sequence similarity.

	AmnoonvirusEGY1	KU751820/TiLV/Israel/Til-4-2011*Index_strian	OM469315/TiLV/India/S27/SRLAAH/2021	OR101705/TiLV/India/KR-7/S2/SRLAAH/2021	OR101707/TiLV/India/CC-7/S4/SRLAAH/2022	OR101706/TiLV/India/RV-7/S3/SRLAAH/2021	OQ437060/TiLV/Hong_Kong/Israel-HK	KU552137/TilapiaVirus/Israrl/AD-2016	ON376578/TiLV/Viet_Nam/HB196-VN-2020	PV356103/TiLV/Colombia/ICA2023	MN939378/TiLV/Bangladesh/BD-2017	BK063216/TiLV-like_virus/Caribbean/Maracas_2015-2	BK063206/TiLV-like_virus/Caribbean/Maracas_2015-1
**AmnoonvirusEGY1**													
**KU751820/TiLV/Israel/Til-4-2011*Index_strian**	**62.03**												
**OM469315/TiLV/India/S27/SRLAAH/2021**	**63.29**	**2.41**											
**OR101705/TiLV/India/KR-7/S2/SRLAAH/2021**	**62.03**	**0.00**	**2.41**										
**OR101707/TiLV/India/CC-7/S4/SRLAAH/2022**	**63.29**	**2.41**	**0.00**	**2.41**									
**OR101706/TiLV/India/RV-7/S3/SRLAAH/2021**	**63.29**	**2.41**	**4.82**	**2.41**	**4.82**								
**OQ437060/TiLV/Hong_Kong/Israel-HK**	**62.03**	**0.00**	**2.41**	**0.00**	**2.41**	**2.41**							
**KU552137/TilapiaVirus/Israrl/AD-2016**	**62.03**	**1.20**	**1.20**	**1.20**	**1.20**	**3.61**	**1.20**						
**ON376578/TiLV/Viet_Nam/HB196-VN-2020**	**62.03**	**2.41**	**2.41**	**2.41**	**2.41**	**4.82**	**2.41**	**1.20**					
**PV356103/TiLV/Colombia/ICA2023**	**63.29**	**1.20**	**2.41**	**1.20**	**2.41**	**3.61**	**1.20**	**2.41**	**3.61**				
**MN939378/TiLV/Bangladesh/BD-2017**	**63.29**	**2.41**	**0.00**	**2.41**	**0.00**	**4.82**	**2.41**	**1.20**	**2.41**	**2.41**			
**BK063216/TiLV-like_virus/Caribbean/Maracas_2015-2**	**65.82**	**15.66**	**16.87**	**15.66**	**16.87**	**18.07**	**15.66**	**15.66**	**15.66**	**16.87**	**16.87**		
**BK063206/TiLV-like_virus/Caribbean/Maracas_2015-1**	**65.82**	**15.66**	**16.87**	**15.66**	**16.87**	**18.07**	**15.66**	**15.66**	**15.66**	**16.87**	**16.87**	**0.00**	

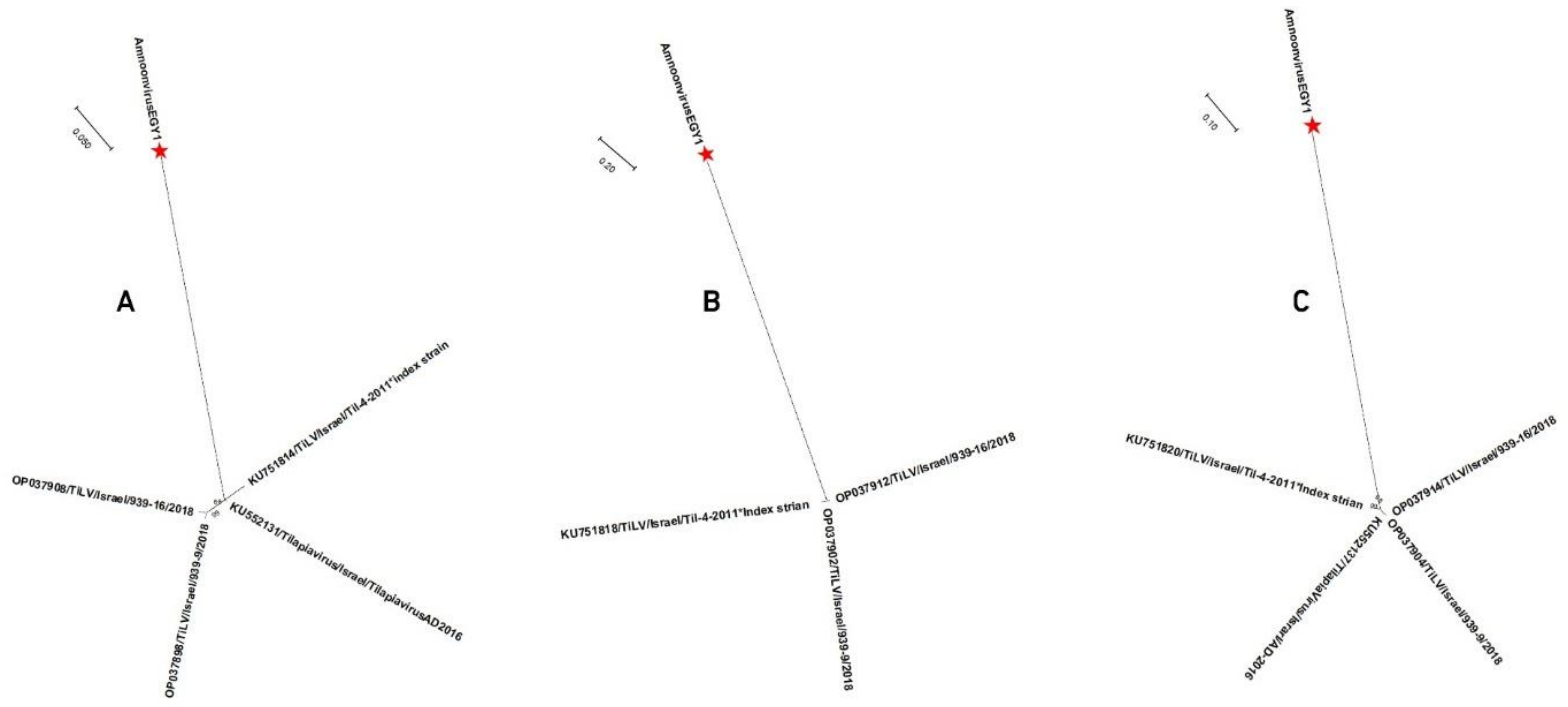

Figure S1. An unrooted maximum likelihood phylograms illustrating the phylogenetic relationships between AmnoonvirusEGY1 nucleotide sequences (indicated by red ★) and TiLV strains from Israel for genomic segments 1, 5, and 7. A. Segment 1 was aligned with four Israeli strains (GenBank Accession Nos. OP037908, OP037898, KU552131, and KU751814). B. Segment 5 was compared with three Israeli strains (OP037912, OP037902, and KU751818). C. Segment 7 was analyzed against four Israeli strains (OP037914, OP037904, KU552137, and KU751820). Each viral isolate is labeled with its GenBank accession number, virus name, country of origin, and isolate designation. Bootstrap values ≥70% are displayed at corresponding nodes to indicate clade support. Scale bars represent the number of nucleotide substitutions per site: 0.050 for panel A, 0.20 for panel B, and 0.10 for panel C.
